# A biocompatible β-cyclodextrin inclusion complex containing natural extracts: a promising antibiofilm agent†

**DOI:** 10.1039/d4na00916a

**Published:** 2025-01-13

**Authors:** Obaydah Abd Alkader Alabrahim, Mostafa Fytory, Ahmed M. Abou-Shanab, Jude Lababidi, Wolfgang Fritzsche, Nagwa El-Badri, Hassan Mohamed El-Said Azzazy

**Affiliations:** a Department of Chemistry, School of Sciences & Engineering, The American University in Cairo AUC Avenue, P.O. Box 74 New Cairo 11835 Egypt hazzazy@aucegypt.edu +202 2615 2559; b Material Science and Nanotechnology Department, Faculty of Postgraduate Studies for Advanced Sciences (PSAS), Beni-Suef University 62511 Beni-Suef Egypt; c Center of Excellence for Stem Cells and Regenerative Medicine, Zewail City of Science and Technology Giza 12578 Egypt nelbadri@zewailcity.edu.eg; d Department of Nanobiophotonics, Leibniz Institute of Photonic Technology Jena 07745 Germany

## Abstract

Biofilms formed by several bacterial strains still pose a significant challenge to healthcare due to their resistance to conventional treatment approaches, including antibiotics. This study explores the potential of loading natural extracts with antimicrobial activities into β-cyclodextrin (βCD) nanoparticles, which are FDA-approved and have superior biocompatibility owing to their cyclic sugar structures, for biofilm eradication. An inclusion complex of βCD carrying *Boswellia sacra* essential oils (BOS) was prepared and characterized with regard to its physicochemical properties, antimicrobial efficacy, and antibiofilm activities. Encapsulation of BOS into βCD significantly enhanced the antimicrobial activity of BOS by 4-fold against Gram-positive (*Staphylococcus aureus* and *Bacillus subtilis*) and by 8-fold against Gram-negative (*Escherichia coli* and *Pseudomonas putida*) bacteria, with minimum inhibitory concentrations ranging from 2.5 to 5 mg mL^−1^. Furthermore, the BOS-βCD complex demonstrated a dual-action against bacterial biofilms where it prevented biofilm formation and disrupted established biofilms. This resulted in a significant reduction in biofilm biomass, with prevention and disruption rates reaching up to 93.78% and 82.17%, respectively. Additionally, the formula revealed an excellent biocompatibility profile with no induction of oxidative stress in human skin fibroblast cells. Our findings suggest that βCD nanoparticles loaded with BOS essential oils hold promise as an effective formula for preventing the formation of bacterial biofilms and combating preformed ones for use in relevant medical applications.

## Abstract 2

Biofilms still pose a significant healthcare challenge, worldwide. βCD nanoparticles loaded with *Boswellia sacra* essential oils (BOS) hold promise for effectively preventing and disrupting bacterial biofilms.

## Graphical abstract

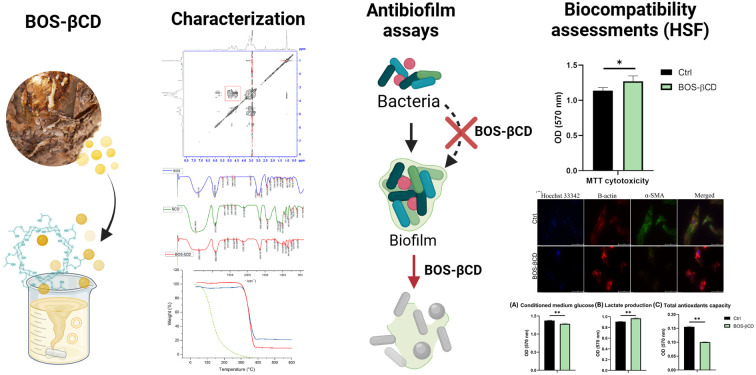

## 1. Introduction

Biofilms are among the major health concerns causing serious bacterial infections which can be developed by several strains of bacteria including *Staphylococcus aureus* (*S. aureus*), *Escherichia coli* (*E. coli*), *Pseudomonas aeruginosa* (*P. aeruginosa*), and *Bacillus cereus* (*B. cereus*).^1,2^ Biofilms are responsible for 1.96 million bacterial infections annually in the United States of America, resulting in higher health costs (>$18B) and more than 260 000 deaths.^3^ Biofilms can be defined as highly organized bacterial communities tightly surrounded by a self-produced protective layer made of extracellular matrix.^1,2^ Therefore, these sorts of biofilms have provoked the formation of multidrug resistant microbes, owing to their capability to maintain a well-protected environment for bacteria with secure support of nutrient supply and provide altered pH medium coupled with greater shear of mechanical forces in the surrounding environment, securing a well-habitable medium for bacteria to combat and develop antibiotic resistance.^1,2,4,5^ Moreover, biofilms can effectively develop resistance against antibiotics, combat host defense system, fight external stressors, and form a prime hindrance in delaying the healing of chronic wounds, establishing a challenging reservoir for harmful and opportunistic pathogens.^1,2,6–8^ About 80% of chronic microbial infections such as otitis, vaginitis, gingivitis, urethritis, conjunctivitis, colitis, prostatitis, and osteomyelitis are attributed to infections developed by bacterial biofilms.^6,7,9^ For instance, *E.coli* were detected in serious urinary tract infections with their capability to develop biofilms.^10,11^ Additionally, *S. aureus* were reported to develop colonies on medical devices developing biofilms and hence causing several nosocomial infections such as osteomyelitis, endocarditis, pneumonia, and sepsis.^12,13^ Thus, targeting infections developed by such biofilms still presents a major challenge which warrants the search of new approaches.

While current strategies exploited to treat biofilms have often involved antibiotics, alternative approaches are urgently needed. Natural extracts, particularly essential oils (EOs), have garnered significant interest attributing to their intrinsic antimicrobial and antibiofilm characteristics.^14–17^ The unique therapeutic benefits of *Boswellia sacra* EOs (BOS) are primarily attributed to their various content of promising bioactive components, including polyphenols, terpenes, and monoterpenoids.^18^ In a previous study, the GC-MS of BOS showed a detailed breakdown of the chemical constituents of BOS and revealed a total of 32 unique compounds that primarily belong to monoterpene in addition to the presence of sesquiterpenes, oxygenated monoterpenes, and esters.^18^ Monoterpenes dominated the GC profile with α-pinene (61.05%) showing the highest percentage, followed by d-limonene, δ-3-carene, camphene, *O*-cymene, and β-pinene.^18^ These compounds exhibited remarkable targeting ability towards the microbial cell membranes enhancing their permeability, thereby impairing critical cellular transport mechanisms and leading to bacterial cell death.^19–22^ By disrupting the lipid layers that protect microbial cells, terpenoids and similar compounds, which are fat-soluble, can more easily penetrate the cell membrane resulting in cell death.^20^ These findings matched the characteristics expected for BOS, aligning with previous research.^23,24^

Furthermore, studies have established insignificant cytotoxic effects and biocompatibility of different extracts of *Boswellia* species on normal cells, such as MCF10-2A (normal breast cells), HEK-293 (human embryonic kidney cells), WI-38 (normal lung cells), and HSF (human skin fibroblast cells).^23,25–27^ However, similar to other EOs, limitations such as poor bioavailability, targeted delivery, and stability hinder their therapeutic potential.^28^

Several nanocarriers were shown as promising platforms to combat bacterial biofilms by facilitating delivery of their encapsulated cargo of antimicrobials into biofilms and reducing their adhesion.^29,30^ Additionally, nanomaterials can effectively eradicate biofilms through induced physical disruption, oxidative stresses, and thermal damage inside the bacterial biofilm.^31–35^ The outstanding physicochemical properties of nanoparticles have facilitated their key role in fighting biofilms due to their unique surface charges, small sizes, and greater biological responses, hence serving as promising multi-functional antimicrobials.^31,32^ Many nanoparticulate-based systems have been remarkably used to encapsulate EOs, augmenting their bioavailability and stability along with facilitating their targeting accessibility to the biofilm site.^29,30^ Nevertheless, the anti-biofilm efficacy and therapeutic effects of EOs loaded into nanocarriers could be substantially improved by promoting their release sustainability and biofilm penetration ability.^36^ Hence, this targeting approach reduces off-target impacts on healthy tissue, thus offering a promising biocompatible strategy and playing a crucial factor in promoting wound healing and tissue regeneration.

Carbohydrate-based nanocarriers particularly those derived from cyclodextrins, have recently shown enhanced therapeutic properties and physicochemical characteristics, offering exceptional biocompatibility and versatility.^14,37^ The polymeric beta-cyclodextrin nanocarriers can be tailored to target the prime components of bacterial biofilms exploiting two major mechanisms. The first mechanism includes the development of molecular inclusion complexation with the essential virulence factor inducing signaling molecules inside the targeted bacterial biofilm. The second mechanism involves disrupting the matrix of the targeted biofilms *via* interactions with polysaccharides.^38,39^ On the other hand, in addition to their accreditation as FDA-approved carriers,^14,18^ beta-cyclodextrins have established superior biocompatibility imparted by their cyclic sugar structures, which is similar to components naturally found in human cells, which augments their further use as biocompatible drug delivery carriers.^40^ Hence, such nanocarriers can be specifically designed to target biofilms while minimizing undesirable effects on normal cells, making them ideal candidates for biocompatible, therapeutic, and wound-healing applications.^29,30,41^

This study aimed to develop and characterize a biocompatible inclusion complex of *Boswellia sacra* essential oils (BOS) loaded with hydroxypropyl-beta-cyclodextrins (BOS-βCD). The study investigated the physicochemical properties, morphology, and entrapment efficiency of the complex. Furthermore, the antimicrobial activity of BOS-βCD against various bacterial strains and biofilms was evaluated and compared to free BOS. Most importantly, *in vitro* cytotoxicity, apoptosis, cytoskeletal arrangement, and reactive oxygen species (ROS) generation were assessed to determine the biocompatibility of BOS-βCD on human skin fibroblast (HSF) cells. Additionally, biochemical analyses, including lactate production, glucose consumption, and total antioxidant capacity, were performed on HSF cells treated with BOS-βCD to further confirm the biocompatibility of BOS-βCD. This comprehensive investigation aimed to establish the BOS-βCD inclusion complex as a safe and effective antibacterial biofilm for potential therapeutic applications.

## 2. Materials and methods

### 2.1. Materials

#### 2.1.1. Chemicals

βCD (2-hydroxypropyl-beta-cyclodextrins) powder and potassium bromide (KBr; FT-IR grade, ≥99%) were obtained from Sigma (Sigma-Aldrich, Co., Germany). Nutrient broth was purchased from Titan Biotech Ltd (Rajasthan, India). Dimethyl sulfoxide was provided by Merck KGaA (Darmstadt, Germany). Acetonitrile (HPLC grade, ≥99.9%) was acquired from VWR Chemicals (Fontenay-sous-Bois, France). *B. sacra* resins were collected in Oman from fully grown *Boswellia sacra* plants.

#### 2.1.2. Bacteria strains

Four bacterial strains: *Escherichia coli* (*E. coli*) (ATCC 25922), *Staphylococcus aureus* (*S. aureus*) (ATCC 25923), *Bacillus subtilis* (*B. subtilis*) (ATCC 6015), and *Pseudomonas putida* (*P. putida*) (ATCC 12633) were acquired from Animal Health Research Institute (Cairo, Egypt). These bacterial cultures were recovered from frozen stocks, inoculated into nutrient broth and incubated for 24 h at 37 °C.

#### 2.1.3. Cell culture

Human skin fibroblasts (HSF; ATCC, Manassas, VA, USA) were maintained in a complete culture medium (CCM) containing DMEM (PAN Biotech, Germany) supplemented with 10% FBS and 1% streptomycin/penicillin and maintained at 37 °C in a humidified 5% CO_2_ incubator. For all experiments, cells received either CCM (control) or treated with β-cyclodextrin inclusion complex containing BOS essential oils at a concentration of 1 mg mL^−1^ in CCM.

### 2.2. Methods

#### 2.2.1. Development of BOS-βCD inclusion complex

The encapsulation of BOS into βCD was carried out by employing the freeze-drying method to form the BOS-βCD inclusion complex, as reported previously with some modifications.^14,18^ The method involved dissolving 10 g of βCD in 50 mL distilled water. Afterwards, 1 g of BOS was added to the solution and the obtained mixture was stirred at 400 rpm in the dark for 48 h at room temperature. Subsequently, a 0.45 micrometer polytetrafluoroethylene (PTFE) filter was utilized to eliminate any unencapsulated BOS. The remaining solution, containing only the BOS-βCD inclusion complex, was frozen at −20 °C for 24 h. Finally, the BOS-βCD inclusion complex was freeze-dried for 48 h. The lyophilized powder of BOS-βCD inclusion complex was then stored in a sealed container within a desiccator.

#### 2.2.2. Morphological examination

The morphological characteristics of βCD particles and BOS-βCD inclusion complex were examined using a Neoscope (JCM-6000 Plus) JEOL Benchtop SEM. For this purpose, small amounts of both samples were fixed on aluminum stubs for which a thin layer of Au was consequently sputtered (15 mA over 5 min).^18,19^

On the other hand, an Ultra-high Resolution Transmission Electron Microscopy (UHR-TEM) was also employed to further assess the morphological features and encapsulation of BOS within BOS-βCD inclusion complex. The UHR-TEM (JEOL JEM-2100 Plus, Tokyo, Japan) was operated at an accelerating voltage of 200 kV. βCD and BOS-βCD were mixed, using minute quantity of each, with distilled water forming corresponding suspensions. Suspensions were then sonicated at 37 °C for 10 min. A droplet of each suspension was placed on a copper grid covered with a thin film of carbon (1 nm). The samples were consequently stained, using 2% uranyl acetate, and dried using a filter paper.^14,42^

#### 2.2.3. Structural examination

Mean size (*Z*-average) and distribution (polydispersity index; PDI) of βCD particles and BOS-βCD inclusion complex were measured using a Zetasizer employing DLS (Malvern Instruments Ltd, Malvern, UK). Suspensions of BOS-βCD and βCD were prepared in distilled water and then measured at 24 °C.^18,43^

To examine the chemical structures of BOS, βCD, and BOS-βCD, a Nicolet 380 FTIR spectrometer (Thermo Scientific, Madison, WI 53719, United States) was employed. The FTIR spectra were recorded in the range of 4000–400 cm^−1^. BOS analysis involved placing a drop of BOS on a KBr disc and aligning it with the infrared beam. Additionally, samples preparation of βCD and BOS-βCD involved their initial mixing with KBr in a 1 : 100 ratio. These mixtures were then pressed into discs using a hydraulic press (15T manual press machine, China).^14,28,44^

To investigate the successful encapsulation of BOS within the cavities of BOS-βCD inclusion complex, ^1^H NMR spectra of BOS, βCD, and BOS-βCD (ESI Fig. S3–S5 and Table 1†) in addition to 2D-HNMR (NOESY) spectrum of BOS-βCD were obtained. To accomplish this, samples were dissolved in DMSO, and an NMR spectrometer was utilized at room temperature (400 MHz, BRUKER BioSpin GmbH, D-76287 Rheinstetten, Germany).^14,45^

Moreover, thermal characteristics of βCD, BOS, and BOS-βCD were assessed. The analysis was conducted using a thermogravimetric analysis device (Labsys Instruments, Paris, France). For this purpose, samples were heated from 25 °C to 600 °C at a constant rate of 10 °C min^−1^ under an atmosphere of nitrogen gas flow. The flow rate of the nitrogen was set at 50 mL min^−1^.^14,28,42^

#### 2.2.4. Entrapment efficiency and drug loading capacity measurements

The amount of BO encapsulated within BOS-βCD inclusion complex was quantified using UV-visible double beam spectrophotometry (Cary 3500 UV-vis Engine, Agilent Technologies) at a wavelength of 270 nm. For this purpose, 5 mg of BOS-βCD inclusion complex was suspended into 5 mL of acetonitrile inside a sealed container (a Falcon tube sealed with a parafilm). The obtained mixture was incubated in the dark at room temperature for 72 h to allow the release of BOS from the inclusion complex of BOS-βCD. A separate calibration curve for BOS was prepared under the same conditions, with concentrations ranging from 3.906 to 125 μg mL^−1^ (*Y* = 0.0031 × *X* + 0.0071; *R*^2^ = 0.9962). Eqn (1) and (2) were then used to calculate entrapment efficiency (% EE) and drug loading capacity (% DL):1
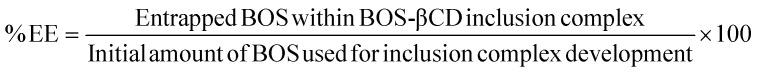
2
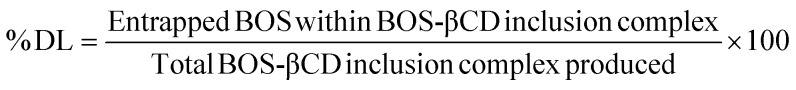


### 2.3. *In vitro* antimicrobial assessments

#### 2.3.1. Antibacterial activity of BOS and BOS-βCD inclusion complex

To determine the minimum inhibitory concentration (MIC) of BOS and BOS-βCD inclusion complex against *E. coli*, *S. aureus*, *P. putida*, and *B. subtilis*, a microdilution assay was performed.^28,46^ The bacterial inoculum was initially prepared by incubating each bacterial culture in nutrient broth overnight until reaching the logarithmic growth phase. The bacterial suspension was then standardized to a concentration of approximately 10^6^ CFU mL^−1^. In a sterile 96-well plate, serial dilutions of BOS and BOS-βCD were prepared to reach final concentrations of 0.625, 1.25, 2.5, 5, 10, and 20 mg mL^−1^. Following this, 10 μL of the bacterial inoculum was added to each well, and the plates were incubated at 37 °C for 24 h to allow bacterial growth. After incubation, 50 μL of Alamar Blue reagent was added to each well to assess bacterial viability based on color change, in which a color-shift from blue to pink indicated active bacterial cells, whereas the absence of color change suggested bacterial inhibition. The MIC was then recorded as the lowest concentration, at which no color change occurred, indicating complete inhibition of bacterial growth. Additionally, free βCD (200 mg mL^−1^) was used as a control along with benzalkonium chloride (1%) that served as a positive control to validate the effectiveness of the observed MIC values.^14,47^

#### 2.3.2. Prevention of biofilm formation and disruption of established biofilm by BOS-βCD inclusion complex

##### 2.3.2.1. Prevention of biofilm formation assay

Bacterial inoculums of *P. putida*, *E. coli*, *S. aureus*, and *B. subtilis* were prepared at a concentration of 10^6^ CFU mL^−1^, in which 100 μL of bacterial inoculum was added to each well of a 96-well plate. Serial dilutions of BOS-βCD were prepared for each bacterial strain examined, covering concentrations both below (½ MIC and ¼ MIC) and above (2 MIC and 4 MIC) the MIC values, determined by the microdilution assay, with 100 μL of each dilution added to the corresponding wells. Free BOS, free βCD, and benzalkonium chloride were treated similarly. The plates were incubated at 37 °C for 24 h at static conditions. After incubation, the contents of the wells were washed three times with sterile PBS to remove non-adherent bacteria. The remaining biofilm was stained with 200 μL of 0.1% crystal violet solution for 20 min at room temperature, followed by washing three times with distilled water to eliminate excess stain. Consequently, the plates were allowed to air-dry completely, and 200 μL of 95% ethanol was then added to each well to solubilize the crystal violet-stained biofilm. The absorbance of each well was measured at 570 nm to quantify the biofilm.^48^

##### 2.3.2.2. Disruption of established biofilm assay

The initial bacterial inoculum of each bacterial strain of *P. putida*, *E. coli*, *S. aureus*, and *B. subtilis* were prepared, followed by overnight incubation at static conditions to facilitate biofilm formation. After incubation, the wells were washed with sterile PBS to remove any planktonic bacteria. Afterward, serial dilutions of the BOS-βCD were added directly to the wells for an additional overnight incubation period. Consequently, the contents of each well were removed, and the wells were washed again with sterile PBS to ensure that only the adherent biofilm, if present, remained. Hence after, 200 μL of 0.1% crystal violet solution was added to each well, following the previously described procedure for staining and quantifying the biofilm. Similar treatment conditions were applied for free BOS, free βCD, and benzalkonium chloride.^48^ The inhibition percentage was calculated using the following equation (eqn (3)):3
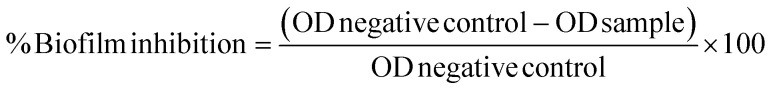


### 2.4. *In vitro* biocompatibility assays

#### 2.4.1. MTT cytotoxicity assay

HSF cells at a density of 5000 cells per well were cultured in two groups including (CCM) as a control group and the other group treated with BOS-βCD at a concentration of 1 mg mL^−1^. 3-(4,5-Dimethylthiazol-2-yl)-2,5-diphenyltetrazolium bromide (MTT) solution (Life Technologies, USA) prepared at a concentration of 5 mg mL^−1^ was added to each well and incubated for 3 h in a 5% CO_2_ humidified atmosphere at 37 °C. Formazan salt was dissolved in anhydrous dimethyl sulfoxide (DMSO) for 15 min using a shaking plate. Optical density (OD) was measured at 570 nm with reference to 630 nm using a FLUOstar Omega-microplate reader (BMG Labtech, Cary, NC, USA).^49,50^

#### 2.4.2. Apoptosis analysis

Annexin V kit (R&D Systems, Minneapolis, USA) was used according to the manufacturer's protocol. Briefly, HSF cells treated with BOS-βCD were washed with PBS, followed by the binding buffer. 1 μL of Annexin V antibody was then added to 100 μL of the binding buffer (1×) and incubated for 30 min. Cells were counterstained with Hoechst 33 342 dye (Molecular Probes, USA) and imaged using a Leica DMi8 inverted fluorescent microscope (Leica Microsystems, Wetzlar, Germany).

#### 2.4.3. Cytoskeleton arrangement analysis

HSF cells were seeded onto glass slides, treated with BOS-βCD, washed, and fixed with 4% paraformaldehyde. Cells were then permeabilized with 1% Triton-X 100 and incubated with 1% bovine serum albumin (BSA) solution to block nonspecific binding of antibodies. Primary Rabbit β-actin antibody (Novus Biologicals, USA) and mouse α-smooth muscle actin (α-SMA) (Invitrogen, USA) were used with secondary goat anti-mouse (Molecular Probes, USA) and goat anti-rabbit (Molecular Probes, USA). Nuclei were counterstained with Hoechst 33 342 (Molecular Probes) for 15 min. Cells were visualized using a Leica DMi8 inverted fluorescent microscope (Leica Microsystems, Wetzlar, Germany).

#### 2.4.4. Oxidative stress assay

Oxidative stress as manifested by reactive oxygen species (ROS) production was evaluated using dihydrorhodamine 123 staining (DHR123, Sigma-Aldrich, St. Louis, MO, USA). After HSF treatment, cells were stained with DHR123 by incubation for 30 min at 37 °C. The cells were then counterstained with Hoechst 33 342 dye, and images were obtained using Leica DMi8 inverted fluorescent microscope.

#### 2.4.5. Metabolic analyses

HSF cells treated with BOS-βCD were analyzed for lactate production using Lactate Plus-Liquizyme enzymatic colorimetric assay kit (Spectrum Diagnostics, Cairo, Egypt). Moreover, glucose enzymatic colorimetric assay kit (Biodiagnostic, Cairo, Egypt) and total antioxidant capacity kit (Biodiagnostic, Cairo, Egypt) were utilized to measure glucose consumption and total antioxidant capacity, respectively. Analyses were done according to manufacturer protocols by measuring the OD at 546 nm for lactate and at 510 nm for both glucose and total antioxidant capacity using a FLUOstar Omega-microplate reader (BMG Labtech, Cary, NC, USA).

### 2.5. Statistical studies

To assess data consistency and identify potential variations, the mean and standard deviation were determined for each measurement set. Three samples were prepared for each formulation. Statistical significance was determined using a *p*-value threshold of 5% or less. ANOVA was employed to detect statistically significant differences between the formulations. ImageJ software was used to determine the particle sizes.

## 3. Results and discussion

### 3.1. BOS-βCD morphological examination

The obtained SEM images (ESI Fig. 1a and b†) depicted the morphology and size variations between βCD and BOS-βCD inclusion complex. While βCD demonstrated large ovoid alongside rectangular-shaped particles, the particles of BOS-βCD inclusion complex were presented with significant reductions in size accompanied by substantial morphological alterations. Remarkably, the particles of BOS-βCD inclusion complex exhibited agglomeration and several aggregates formation, indicating an interaction between larger and smaller particles occurred. Such observations suggested the potential complexation formation of BOS-βCD inclusion complex, possibly involving an amorphous product interacting with other components in the established complex. These findings are consistent with previous research.^18,51^

**Fig. 1:**
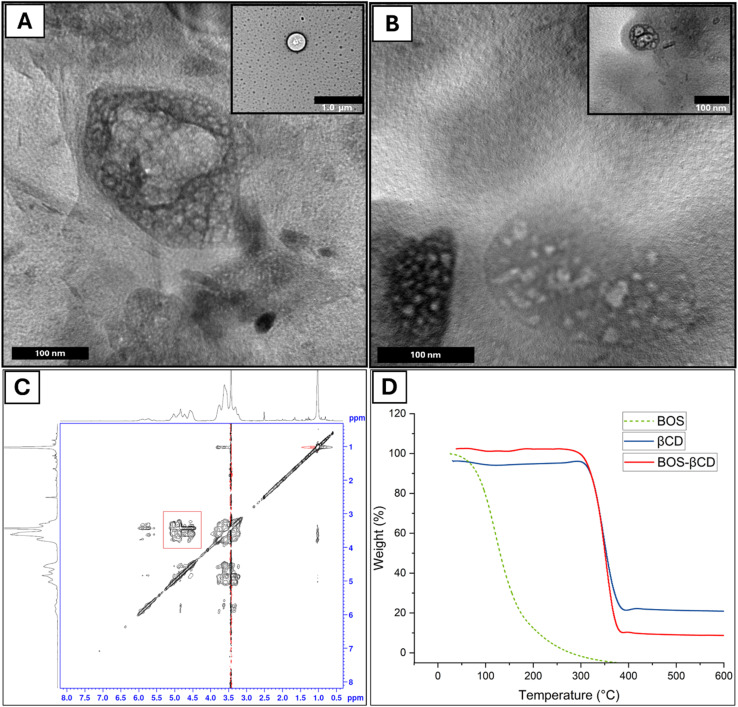
UHR-TEM images for (A) βCD and (B) BOS-βCD inclusion complex providing insights into their structures, shape, and encapsulation confirmation. Particles of both βCD and BOS-βCD appeared as round vesicles surrounded with thin membranes. More importantly, particles of BOS-βCD inclusion complex displayed aggregates, and both samples contained various micellar structures, attributing to cyclodextrins' self-assembly. 2D-NOESY spectrum (C) of BOS-βCD in DMSO illustrates the interactions between the BOS protons and the βCD protons within the BOS-βCD. TGA curves (D) of BOS, βCD, and BOS-βCD inclusion complex.

Furthermore, UHR-TEM images (Fig. 1A and B) were examined to assess the encapsulation of BOS within the inclusion complex of BOS-βCD and to investigate the structural and morphological features of βCD and BOS-βCD. Notably, βCD displayed round vesicles (diameters: 82.54–412.84 nm) with larger micellar structures often formed. Conversely, the particles of BOS-βCD inclusion complex exhibited nearly spherical vesicles (diameters: 14.53–83.19 nm), with a thin membrane surrounding the encapsulated BOS, with a strong tendency to aggregate showing larger agglomerates (diameters: 141.53–401.61 nm), in which larger particles attracted smaller ones. These observations suggest a successful BOS encapsulation within the cavities of βCD forming their consequent BOS-βCD inclusion complex. On the other hand, both samples depicted several micellar structure formations that can be explained with the use of distilled water to dissolve both samples prior to their preparation for imaging, in which cyclodextrins manage to self-assemble under such conditions. Similar results were reported in previous studies.^14,18,43^

Significant differences in the particles size and distribution could be noticed among βCD and BOS-βCD inclusion complex. βCD exhibited a highly polydisperse system, with a PDI of 1.0 (>0.7), and a significantly heterogeneous particles size distribution with a *Z*-average of 1038.53 ± 221.26 nm. Conversely, BOS-βCD inclusion complex exhibited a PDI value of 0.38 ± 0.09 and *Z*-average of 323.37 ± 21.85 nm, reflecting a more uniform system with consistent particles size distribution. These findings can be explained by the well-known tendency of cyclodextrins, including βCD, and their developed inclusion complexes to self-assemble in water forming larger aggregates such as micelles, helping to dissolve lipophilic substances.^14,42,52^

### 3.2. FTIR analysis

FTIR spectra were obtained for investigating the BOS encapsulation within BOS-βCD, inclusion complex formation, and structural characteristics presented for BOS, βCD, and BOS-βCD inclusion complex (ESI Fig. S2†). BOS FTIR spectrum displayed characteristic peaks corresponding to O–H stretching (3435.1 cm^−1^), –CH_2_^−^ group stretching (2926.3 cm^−1^), C–H stretching (2716.7 cm^−1^), H–O–H bending (1668.5 cm^−1^), C–H scissoring (1453.9 cm^−1^), C–O stretching (1364.7 cm^−1^), C–O–C stretching vibrations (1249.5 cm^−1^ and 1076.3 cm^−1^), and C–H bending of aromatics (882.7 cm^−1^ and 814.5 cm^−1^). Nevertheless, the spectrum of βCD showed prominent bands for O–H stretching (3463.7 cm^−1^), –CH_2_^−^ group stretching (2912.2 cm^−1^), C–H stretching (2667.1 cm^−1^), H–O–H bending (1640.5 cm^−1^), C–H vibration (1443.7 cm^−1^), and symmetric and asymmetric C–O–C stretching vibrations (1146.3 cm^−1^ and 1011.9 cm^−1^). Interestingly, the FTIR spectrum of BOS-βCD inclusion complex showed that the characteristic peaks of BOS were obscured by more intense βCD's absorption bands, suggesting potential encapsulation of BOS within the βCD cavities and potential interactions occurred outside these cavities. Additionally, the shifts and broadening of O–H bands observed in BOS-βCD spectrum support this notion, in which inter-molecular hydrogen bonds might be occurred. Also, the broader –CH_2_ band further suggests the successful incorporation of the hydrophobic components of BOS into the BOS-βCD inclusion complex cavities. Overall, the findings presented herein confirm successful BOS encapsulation and formation of stable BOS-βCD complex. Similar results were previously reported.^14,19,44,45^

### 3.3. 2D-NMR NOESY spectroscopy

The encapsulation of BOS within the cavities of BOS-βCD inclusion complex was investigated, employing ^1^H NMR spectra of BOS, βCD, and BOS-βCD (ESI Fig. S3–S5 and Table 1†) in addition to 2D-HNMR (NOESY) spectrum of BOS-βCD (Fig. 1C). The 2D-NOESY spectroscopy method employs the Nuclear Overhauser Effect (NOE) to signify potential interactions imparted by the close proximity of BOS components (the guest molecule) and βCD (the host) within the developed BOS-βCD inclusion complex. While the NMR spectroscopy can refer to the successful formation of BOS-βCD inclusion complex, NOE provides a deeper understanding, in which it can detect the transfer of spin polarization established between nearby atoms in separate populations (BOS and BOS-βCD), reflecting interactions occurred within a short distance (around 0.4 nm).^14,18,53,54^ The indicated interactions are observed as crossed peaks (NOE correlations) in a 2D-NMR NOESY spectrum (Fig. 1C). Notably, the figure showed a significant correlation, imparted by the spatial proximity, between BOS's protons and specific βCD's protons (H3 and H5) within the inclusion complex BOS-βCD. These findings support the successful encapsulation of BOS components within the cavities of the obtained inclusion complex of BOS-βCD which further agrees with previous reports.^14,18,55–57^

### 3.4. TGA analysis

TGA curves for BOS, βCD, and BOS-βCD were obtained (Fig. 1D), depicting the weight loss as a function of temperature. For the TGA curve of BOS, the curve exhibited a rapid weight loss yet a well-stable thermal profile of BOS with 85.14% of total BOS loss, from 53.61 °C to 199.37 °C. The rapid loss of BOS is attributed to its high volatile nature and the rapid decomposition rates of EOs in general.^14,18^ On the other hand, the TGA curve of βCD exhibited an insignificant weight loss up to 297 °C, whereas a significant loss of 71.65% could be detected between 297 °C and 396 °C which can be explained by the thermal decomposition of the βCD molecules. More importantly, BOS-βCD curve showed a very similar thermal behavior to the TGA curve of βCD with some key differences. Both showed minimal weight loss initially, with a small amount of weight loss observed (1.5% at 98.9 °C) in the BOS-βCD inclusion complex owing to the evaporation of the water molecules. Additionally, the major weight loss (87.17%) happened at a slightly lower temperature range (288 °C to 385 °C) compared to βCD alone, indicating some influence from the BOS component. Thus, the encapsulation of BOS into BOS-βCD inclusion complex remarkably enhanced its thermal stability which was evidenced by the significant shift shown in the temperature at which the weight loss of the inclusion complex could be observed in comparison to BOS alone. These results are well matched with the previously published reports.^44,58^

### 3.5. Entrapment efficiency and drug loading capacity of BOS-βCD inclusion complex

BOS-βCD inclusion complex demonstrated excellent encapsulation efficiency (% EE) of 94.78 ± 1.87% and drug loading capacity (% DL) of 8.62 ± 0.17%. The exceptional % EE can be attributed to extended complexation process conducted alongside with the well-protected conditions applied on the obtained solution of the inclusion complex and drying process, positively affecting the encapsulation portion of the BOS, as previously reported.^14,59^ These findings are supported by previous reports which showed strong affinities of specific components presented in the GC-MS profile of BOS, such as α-pinene and limonene, to the βCD cavities.^14,18,60^ On the other hand, the determined %DL (8.62 ± 0.17%) of the obtained inclusion complex aligns with the previous reports established on similar complexes and thus validating the presented findings.^14,42^

### 3.6. *In vitro* antimicrobial and antibiofilm activities

#### 3.6.1. Antibacterial activity of BOS and BOS-βCD inclusion complex

The findings of this study demonstrate varied antimicrobial effects of βCD, BOS, and BOS-βCD inclusion complex on a range of bacterial pathogens, highlighting the improved efficacy of the complexed form. Free βCD exhibited no antibacterial activity against *E. coli*, *S. aureus*, *P. putida*, and *B. subtilis*, excluding any synergistic antimicrobial effects might be imparted by βCD particles. The absence of antibacterial effect of βCD is likely due to its non-toxic and biocompatible nature, which is known to have minimal direct impact on bacterial cell viability.^18,61^ BOS alone exhibited moderate activity with an MIC of 20 mg mL^−1^ across all tested bacteria. This moderate efficacy might have resulted from the hydrophobic nature of BOS that can disrupt bacterial cell membranes.^18,62^ However, the antimicrobial potential of BOS is limited due to reduced solubility and bioavailability in its free form.^18,62^ BOS-βCD inclusion complex, on the other hand, exhibited significantly enhanced antimicrobial activity with MIC values of 2.5 mg mL^−1^, against *P. putida* and *E. coli*, and 5 mg mL^−1^, against *S. aureus* and *B. subtilis*. The increased efficacy of BOS-βCD complex might be attributed to the improved solubility and stability of BOS upon its encapsulation into βCD, facilitating greater interaction with bacterial membranes and enhanced cell permeability.^14,18^ Gram-negative bacteria like *P. putida* and *E. coli*, which possess an outer membrane that often restricts penetration of hydrophobic substances, might be more susceptible to the complexed form due to the better penetration, solubility, and sustained release imparted by βCD complex.^63,64^ Additionally, *S. aureus* and *B. subtilis* were susceptible to higher MIC values than Gram-negative strains, likely due to their thicker peptidoglycan layer which may still hinder the efficacy of BOS in its non-complexed form.^63,64^ Our results align with the findings shown by Arrais *et al.*,^65^ in which an inclusion complex of *Thymus vulgaris*-βCD was developed demonstrating an increased antimicrobial efficacy against both *P. aeruginosa* and *S. aureus*, compared to free EO of *T. vulgaris*, owing to the enhanced solubility and stability imparted by the inclusion complex developed.^65^ The MIC values of βCD and BOS, both free and encapsulated into BOS-βCD inclusion complex, against different bacterial strains are summerized in Table 1.

**Table 1:** MIC values (mg mL^−1^) of βCD and BOS, both free and encapsulated into BOS-βCD inclusion complex. MIC value represents the lowest concentration at which no color change was observed, upon performing Alamar Blue test, indicating complete inhibition of bacterial growth (*n* = 5). MIC values of BOS-βCD were determined based on the encapsulation efficiency of BOS

	*P. putida*	*S. aureus*	*E. coli*	*B. subtilis*
βCD	>200	>200	>200	>200
BOS	20	20	20	20
BOS-βCD	2.5	5	2.5	5

#### 3.6.2. Biofilm prevention and disruption induced by BOS-βCD inclusion complex

**Fig. 2:**
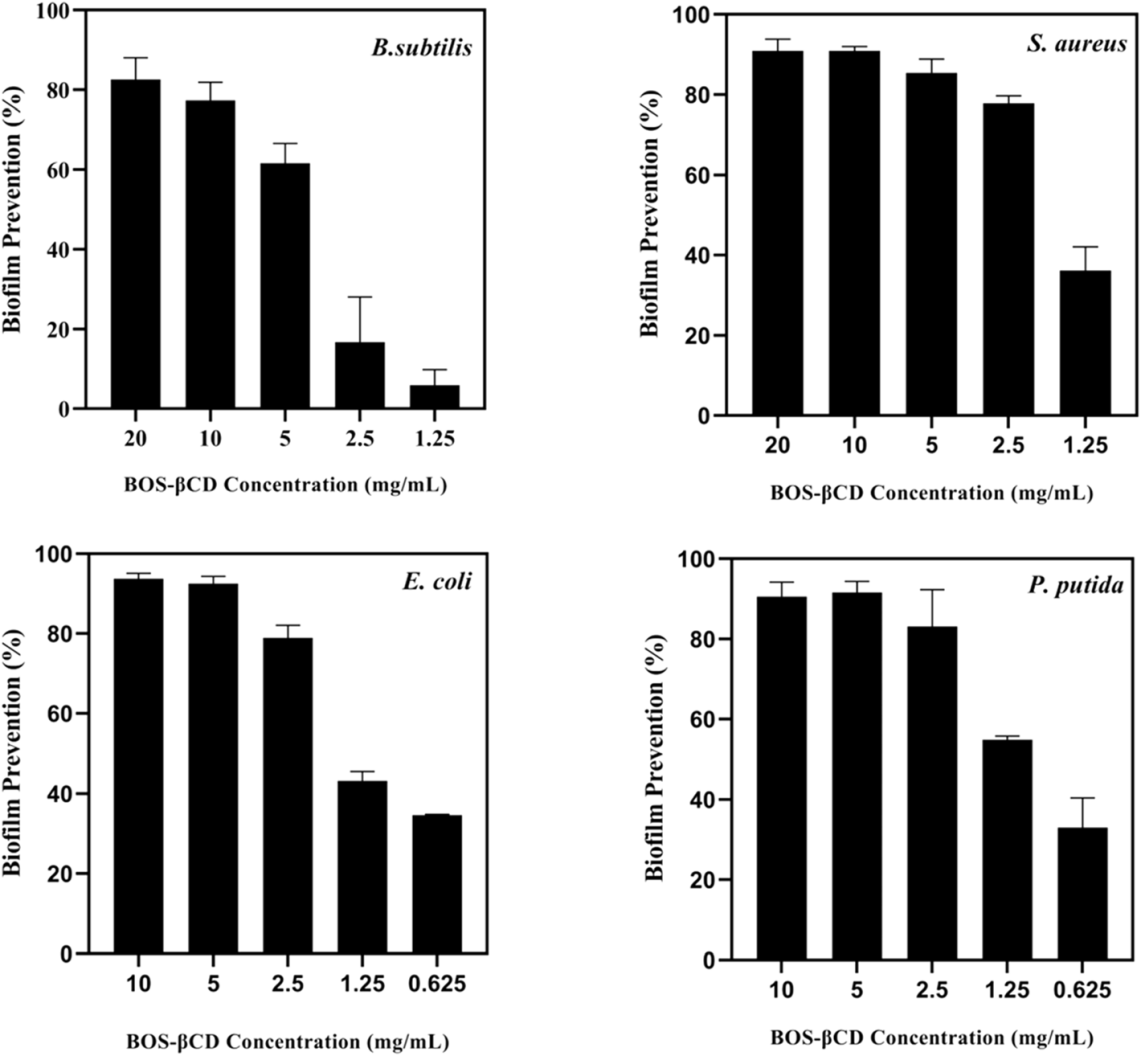
Prevention of biofilm formation of *B. subtilis*, *S. aureus*, *E. coli*, and *P. putida* following treatment with five different concentrations of BOS-βCD complex. These included the predetermined MIC concentration against each bacterial strain in addition to two concentrations above the MIC value (2 MIC and 4 MIC) and another two concentrations below the MIC (½ MIC and ¼ MIC). Data are presented as mean (*n* = 5) ± SD (error bars).

**Fig. 3:**
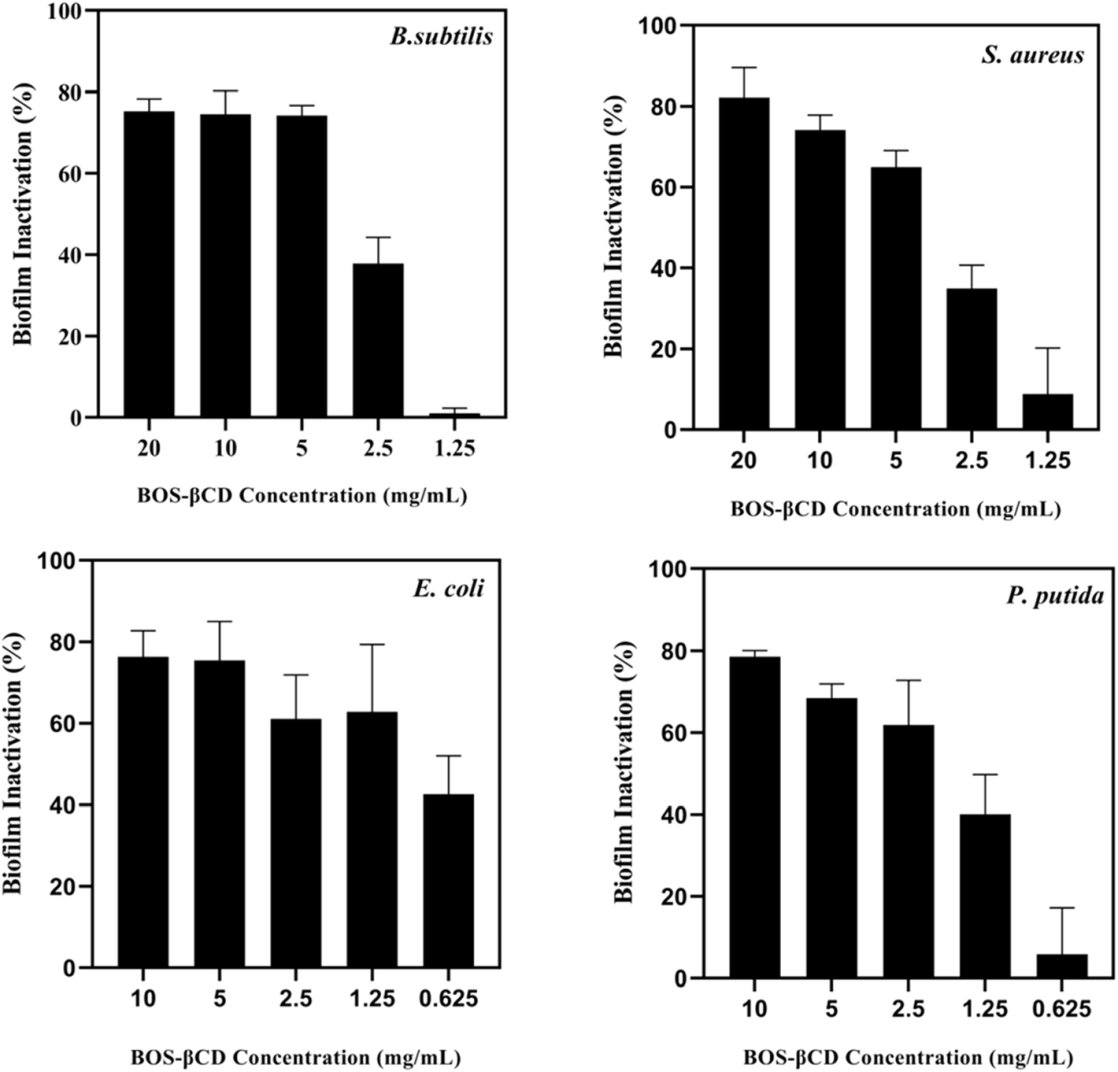
Biofilm disruption of *B. subtilis*, *S. aureus*, *E. coli*, and *P. putida* following treatment with different concentrations of BOS-βCD complex. These included the predetermined MIC concentration (against each bacterial strain) in addition to two concentrations above the MIC value (2 MIC and 4 MIC) and another two concentrations below the MIC value (½ MIC and ¼ MIC). Data are presented as mean (*n* = 5) ± SD (error bars).

Following the determination of the MIC values of BOS-βCD complex against the bacterial strains tested (*P. putida*, *E. coli*, *S. aureus* and *B. subtilis*), the complex efficacy in formation prevention and disruption of biofilms was assessed employing five different concentrations against each bacterial strain (*i.e.*, predetermined MIC concentration against each bacterial strain in addition to two concentrations above the MIC value (2 MIC and 4 MIC) and another two concentrations below the MIC (½ MIC and ¼ MIC), as seen in Fig. 2 and 3).

##### 3.6.2.1. Prevention of biofilm formation

For the biofilm prevention studies, the BOS-βCD complex showed varying efficacy across the examined strains (Fig. 2), reflecting how bacterial structure and physiology influence the biofilm formation. In particular, for *S. aureus*, the BOS-βCD demonstrated a significant biofilm prevention (90.88 ± 2.97%) at 20 mg mL^−1^, securing an effective hinder of the bacterial adhesion and aggregation that are key steps in biofilm formation.^66^ At lower concentrations, the prevention rates remained substantial, showing 90.90 ± 1.11% at 10 mg mL^−1^ and 85.40 ± 3.46% at 5 mg mL^−1^, indicating the ability of BOS-βCD to maintain its effectiveness even as concentrations decreased. This correlates well with the predetermined MIC findings, suggesting that concentrations at and above the MIC value are crucial for optimal biofilm prevention.^67^ Similarly, *B. subtilis* exhibited 82.55 ± 5.48% of its biofilm prevention at 20 mg mL^−1^, with notable reductions seen at complex concentrations of 10 mg mL^−1^ (77.31 ± 4.56%) and 5 mg mL^−1^ (61.66 ± 4.94%), reflecting a potential enhancement in the inclusion complex penetration through the peptidoglycan layer of the Gram-positive cell wall, disrupting the initial adhesion processes essential for biofilm development.^68^ On the other hand, the BOS-βCD complex reached 90.60 ± 3.53% prevention of *P. putida* biofilm utilizing a complex concentration of 10 mg mL^−1^, coupled with persistent efficacy shown at lower concentrations with prevention of 91.57 ± 2.80% at 5 mg mL^−1^ and 83.11 ± 9.20% at 2.5 mg mL^−1^. The enhanced solubility of BOS EO due to βCD might have allowed better penetration through the bacterium outer membrane, effectively disrupting adhesion mechanisms. This aligns with the predetermined MIC, reinforcing the notion that maintaining concentrations above the MIC is vital for combating biofilm formation.^69,70^ Furthermore, 93.78 ± 1.3% of *E. coli* biofilm was prevented at 10 mg mL^−1^ of BOS-βCD while a remarkable prevention of 92.51 ± 1.8% was achieved at 5 mg mL^−1^, reflecting the BOS-βCD effectiveness in inhibiting biofilm formation even with robust biofilm matrices.^69,70^ The trend observed here emphasizes that while prevention rates slightly decrease with lower concentrations of the complex utilized, they remain significant enough to suggest that the BOS-βCD complex effectively disrupts early biofilm formation stages, consistent with the observed MIC results. Notably, neither free βCD nor free BOS alone achieved this level of biofilm inhibition.

##### 3.6.2.2. Disruption of established biofilm

The biofilm disruption/inactivation potential of BOS-βCD was examined against the same bacterial strains (*P. putida*, *E. coli*, *S. aureus* and *B. subtilis*), as shown in Fig. 3. For this purpose, the bacterial strains were cultured overnight for biofilm formation, then serial dilutions of BOS-βCD were added to examine their effects on the already formed biofilm. The individual compounds of free βCD and BOS EO could not reveal any inhibitory effect against the developed biofilms. For *B. subtilis*, the BOS-βCD achieved an inactivation effect of 75.26 ± 2.99% against the biofilm formed at 20 mg mL^−1^, decreasing slightly to 74.52 ± 5.74% at 10 mg mL^−1^ and 74.22 ± 2.41% at 5 mg mL^−1^. At concentrations below the predetermined MIC value (<5 mg mL^−1^), the biofilm inhibition effect dropped to 37.82 ± 6.43%, reflecting a significant reduction in the complex efficacy as the concentration decreased below the MIC value. The strong inhibition at higher concentrations suggests that BOS-βCD effectively disrupts the *B. subtilis* biofilm matrix, likely through terpene-mediated (present in BOS EO) disruption of cell walls and inhibition of quorum sensing pathways, both of which are critical for biofilm formation in Gram-positive bacteria.^71^ For *S. aureus*, the biofilm inhibition reached 82.17 ± 7.35% and 74.13% ± 3.73 at 20 mg mL^−1^ and 10 mg mL^−1^, respectively, whereas at 5 mg mL^−1^, the inhibition slightly decreased to 64.94 ± 4.13%. Conversely, the activity sharply declined at concentrations below 5 mg mL^−1^ (below MIC), with an inhibition of 34.94 ± 5.70% at 2.5 mg mL^−1^ and only 8.81 ± 11.38% at 1.25 mg mL^−1^. These findings suggest that BOS-βCD, at higher concentrations, could effectively impact the *S. aureus* biofilm structure by disrupting cellular adhesion and protein synthesis.^18,72^ For *E. coli*, biofilm inhibition rates reached 76.36 ± 6.41% at 10 mg mL^−1^ and 75.48 ± 9.48% at 5 mg mL^−1^. As concentration dropped to 2.5 mg mL^−1^ (MIC), the biofilm inhibition decreased to 61.15% ± 10.71, while maintaining a similar inhibition rate of 62.86 ± 16.55% at 1.25 mg mL^−1^. Below this, at 0.625 mg mL^−1^, the inhibition dropped to 42.56 ± 9.45%. These findings indicate that the effectiveness of BOS-βCD in penetrating the cell wall of *E. coli* might be correlated to the enhanced solubilization and targeting ability of BOS EO inside the βCD complex to reach intracellular targets involved in biofilm regulation.^73^ Finally, the BOS-βCD exhibited the highest biofilm inhibition effect against *P. putida* at 10 mg mL^−1^, reaching 78.61 ± 1.42%, which decreased to 68.40 ± 3.45% at 5 mg mL^−1^. At 2.5 mg mL^−1^ (MIC), the inhibition was 61.85 ± 10.95%, while a concentration of 1.25 mg mL^−1^ resulted in a significant drop to 40.06 ± 9.67%, and the lowest concentration of 0.625 mg mL^−1^ showed only 5.86 ± 11.33% inhibition. These findings suggest that the BOS-βCD effectively impacted the biofilm formed by *P. putida*, likely by interfering with the biofilm-promoting factors. The current results are consistent with previously published studies, in which Kfoury *et al.* reported that the encapsulation of EOs in cyclodextrins could increase their aqueous solubility up to 16-fold and reduce their photodegradation rates (augmenting stability) up to 44-fold. These improvements led to enhanced antimicrobial and antibiofilm activities.^73^ Moreover, Jaroš *et al.* demonstrated that the boswellic acids derived from BOS EO could significantly inhibit the biofilm formed by three bacterial strains (*Staphylococcus epidermidis*, *Enterococcus faecalis*, and *E. coli.*), making them potent agents against bacterial infections.^74^

### 3.7. Biocompatibility assessments

#### 3.7.1. Cytotoxicity evaluation of BOS-βCD on HSF normal cells

To evaluate the potential cytotoxicity of the BOS-βCD inclusion complex on normal cells, we investigated its effect on apoptosis. HSF cells treated with the BOS-βCD inclusion complex demonstrated resistance to apoptosis, as evidenced by a significant reduction in Annexin V staining compared to the control group. Annexin V, a well-established marker of apoptosis, was employed to assess cell viability. Immunostaining for Annexin V in the control group revealed no fluorescence, confirming the healthy, unstressed, and non-apoptotic state of the untreated HSF cells. Similarly, no Annexin V staining was observed in the BOS-βCD-treated group, indicating that the inclusion complex did not induce apoptosis in these cells. These findings strongly support the biocompatibility and safety profile of the BOS-βCD inclusion complex in normal cells.

**Fig. 4:**
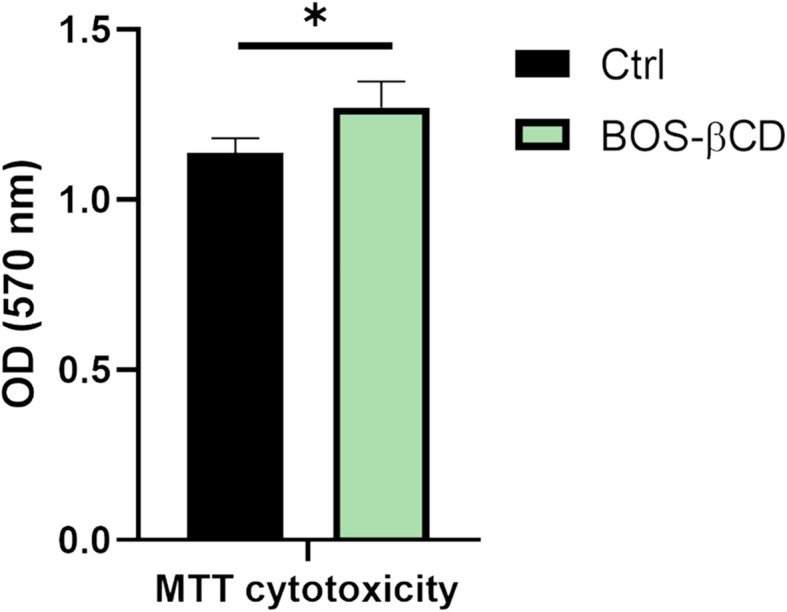
MTT cytotoxicity analysis demonstrated a significant enhancement (*p* < 0.05) of HSF metabolic activity upon treatment with BOS-βCD inclusion complex. Values represent the mean ± SE obtained from three independent experiment measurements. The *p* values were recorded comparing the control group. * Indicates significant statistical difference (*p* < 0.05).

On the other hand, the MTT cytotoxicity assay (Fig. 4) demonstrated a significant enhancement (*p* < 0.05) of HSF metabolic activity upon treatment with BOS-βCD. Hence, these findings suggest a favorable safety profile for BOS-βCD when applied to normal HSF cells. Assays for apoptosis resistance and cellular metabolic activity revealed that the BOS-βCD did not induce programmed cell death and might even have stimulated metabolic processes in HSF cells.

Findings of this study are consistent with the results published by Singh *et al.* which evaluated the safety of repeated oral administration of BOS to rats over 90 days which suggested that BOS is safe in rats up to a dose of 500 mg per kg body weight.^75^ Moreover, Bian *et al.* findings demonstrated that the formation of a naringin-βCD inclusion complex and incorporating it in chitosan hydrogel for wound healing applications exhibited biocompatibility in MTT assay against fibroblast L929 cells.^76^ Another research group reported that cannabidiol-βCD inclusion complex enhanced the properties of PVA-borax hydrogels for wound management applications, as it exhibited antibacterial and antioxidant properties without inducing cytotoxicity towards RAW 264.7 cells.^77^ These results further suggested that βCD, even when complexed with cannabidiol, naringin, or BOS, did not lead to apoptosis in cells. The BOS-βCD inclusion complex could present a promising platform for many biomedical applications, including drug delivery, based on its remarkable antimicrobial profile and established biocompatibility.

#### 3.7.2. BOS-βCD inclusion complex regulates the cytoskeleton arrangement of HSF cells

**Fig. 5:**
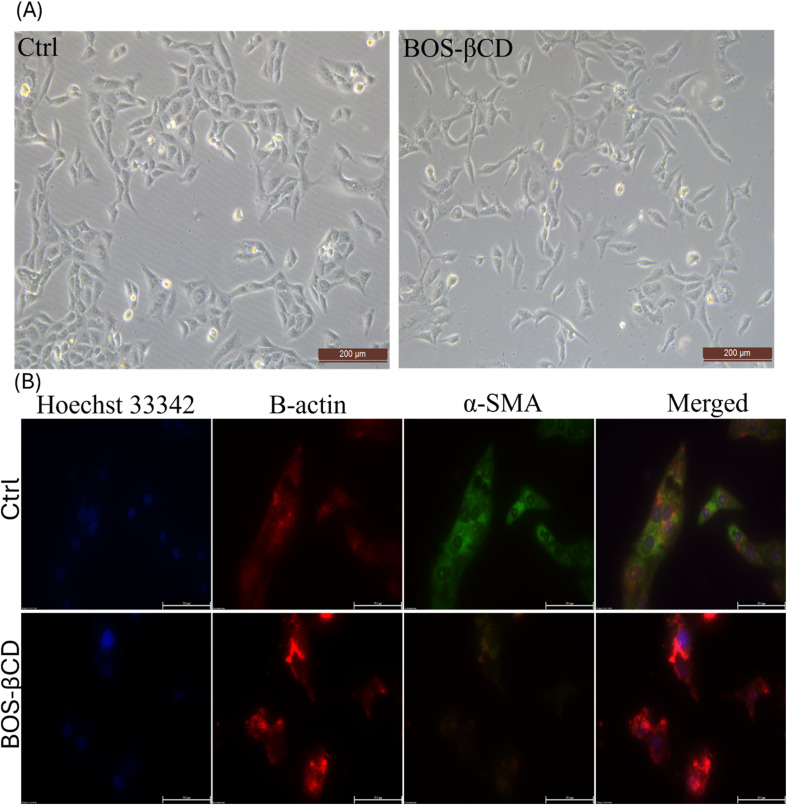
Cellular morphology analysis for HSF as a control group and the treated one with BOS-βCD. (A) Bright-field examination of HSF cells treated with BOS-βCD. (B) Immunofluorescence staining of cytoskeletal β-actin and α-SMA in HSF cells.

To assess the potential disruption of the cytoskeleton induced by BOS-βCD, cellular morphology and protein expression patterns were evaluated. Microscopic observation of HSF cells (Fig. 5A) revealed no apparent changes in the overall cell shape or size between the treated and control groups. The lack of significant morphological changes observed under microscopy verifies the safety profile of BOS-βCD.

Immunofluorescence staining of β-actin, a major cytoskeletal component, did not reveal any significant alterations in the organization of the filamentous network (Fig. 5B). Conversely, α-SMA, a marker for contractile stress fibers, was downregulated in treated cells compared to control. The observed decrease in α-SMA suggests potential modulation of cytoskeleton that could affect cellular contractility.^78^ Interestingly, this decrease in α-SMA coincides with the enhanced metabolic activity observed by the MTT assay. This could be indicative of a shift towards a more proliferative phenotype in BOS-βCD treated cells. Proliferating cells generally exhibit lower levels of α-SMA as they prioritize growth and division over contractility.^78,79^ These effects align with the requirements that may affect the wound healing process, where reduced contractility allows cells to migrate and participate in tissue repair, and proliferative cells contribute to faster wound closure and tissue regeneration. Therefore, modulation of cellular behavior by BOS-βCD supports their safety profile by promoting both cell migration and proliferation.^80–82^ Further investigation is needed to confirm and elucidate the specific mechanisms by which BOS-βCD influences α-SMA expression and cellular proliferation.

#### 3.7.3. BOS-βCD enhances HSF metabolism

Ensuring that BOS-βCD does not induce excessive oxidative stress or metabolic dysfunction is essential for confirming the safety profile of BOS-βCD. Hence, to assess the safety of the inclusion complex, we evaluated its potential to induce oxidative stress. Reactive oxygen species (ROS) are generated as byproducts during cellular metabolism and are tightly regulated under physiological conditions by antioxidant systems. Elevated ROS levels can lead to oxidative stress, causing cellular damage and death. To quantify ROS production, we employed DHR 123 staining. DHR 123 is a cell-permeable dye that fluoresces upon oxidation by ROS. Our results demonstrated no significant increase in ROS production in HSF cells treated with the BOS-βCD inclusion complex compared to the control group at a concentration of 1 mg mL^−1^, suggesting that BOS-βCD did not induce oxidative stress and was safe for HSF cells.

**Fig. 6:**
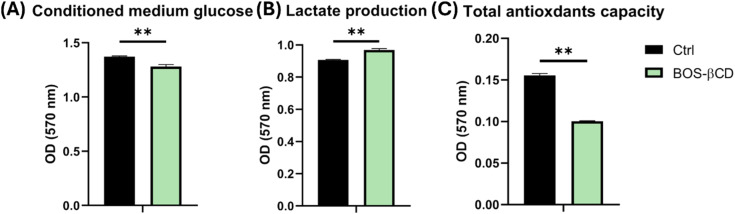
Analysis of HSF metabolism. (A) Analysis of HSF glucose uptake. (B) HSF lactate production assessment. (C) Analysis of HSF total antioxidant capacity. Results values represent the mean ± SE obtained from three independent experiment measurements. The *p* values were recorded as compared to the control group. Significant differences *p < 0.05, **p < 0.01.

Also, the antioxidant capacity in the HSF-treated cells was significantly reduced as compared to the control cells (Fig. 6B). Similarly, the study of Bayiha *et al.* demonstrated that budesonide-βCD inclusion complex offered protective effects against ROS generation, decreased PI3K/Akt activation, and reduced phosphorylated/unphosphorylated HDAC2, highlighting the protective and anti-cytotoxic activity of βCD.^83^

Glucose consumption and lactate production of HSF cells were further measured to assess the impact of BOS-βCD on HSF cell metabolism. HSF cells treated with BOS-βCD resulted in a significant increase in cellular glucose uptake compared to control cells, as evidenced by lower glucose levels in the conditioned medium (Fig. 6A). This elevated glucose uptake was accompanied by enhanced lactate production in the treated cells (Fig. 6B). These findings suggest that BOS-βCD treatment might stimulate the metabolic activity of HSF cells, potentially shifting them toward a more glycolytic phenotype.

The enhanced metabolic activity observed on BOS-βCD treated cells likely plays a role in the potential increase in proliferation.^84^ Increased glucose uptake and lactate production suggest a shift towards a more glycolytic metabolic phenotype, which is a hallmark of rapidly dividing cells.^85^ This metabolic reprogramming allows proliferating cells to generate the building blocks and energy necessary for rapid growth and division.^86,87^ Furthermore, the observed decrease in antioxidant capacity, as indicated in Fig. 6C, could be a consequence of this metabolic shift, or it could represent a separate effect of BOS-βCD inclusion complex. These findings highlight the potential role of BOS-βCD to optimize their use in relevant biomedical applications by supporting energy metabolism, regulating oxidative stress, and enhancing immune responses, thereby promoting effective tissue regeneration. The potential long-term effects of BOS-βCD on HSF cell metabolism and proliferation require further investigation.

## 4. Conclusions

Biofilms pose a significant challenge due to their resistance to conventional treatments. *Boswellia sacra* essential oils (BOS) have shown significant antibacterial, anticancer, and antioxidant properties. This study explored the encapsulation of BOS into hydroxypropyl-beta-cyclodextrin (βCD), an FDA-approved polymeric carrier, as a potential approach for targeting bacterial infections related to biofilms. BOS-βCD demonstrated remarkable physicochemical properties, high encapsulation efficiency (94.78 ± 1.87%), and superior antimicrobial activity compared to free BOS by 4-fold against *S. aureus* and *B. subtilis* and by 8-fold against *E. coli* and *P. putida*, with minimum inhibitory concentrations ranging from 2.5 to 5 mg mL^−1^. Furthermore, BOS-βCD effectively inhibited initial bacterial adhesion, reaching a prevention effect of 93.78 ± 1.3%, and disrupted established biofilms, achieving an inhibition rate of 82.17 ± 7.35%, highlighting its potential as a dual-action agent for biofilm management. These findings demonstrate the potential of BOS-βCD as a promising agent to combat biofilm-associated infections by both preventing biofilm formation and disrupting established biofilms. The BOS-βCD complex also exhibited noticeable biocompatibility, where neither cytotoxicity, oxidative stress, nor disruption of the cytoskeletal protein β-actin, were observed in HSF cells treated with BOS-βCD. The complex also enhanced metabolic activity and proliferation of HSF cells.

In conclusion, BOS-βCD complexes have the potential of effectively preventing the formation of bacterial biofilms as well as preformed biofilms developed by four common bacterial strains and combating other biofilm-associated healthcare challenges. The combination of potent antimicrobial activity and excellent biocompatibility supports the use of BOS-βCD as an antibacterial and an antibiofilm dual-agent which could potentially improve outcomes for patients affected by biofilm-induced infections.

## Data availability

The data supporting this article have been included as part of the ESI.†

## Conflicts of interest

All authors declare no competing interests.

## Supplementary Material

NA-007-D4NA00916A-s001
