# Supplementary material for: A biocompatible β-cyclodextrin inclusion complex containing natural extracts: a promising antibiofilm agent
Source: Nanoscale Adv. 2025 Jan 13;7(5):1405–20. doi: 10.1039/d4na00916a (PMC11748956; doi:10.1039/d4na00916a)
Supplement: NA-007-D4NA00916A-s001 [file NA-007-D4NA00916A-s001.pdf]

Supplementary Information (SI) for Nanoscale Advances.
This journal is © The Royal Society of Chemistry 2025

# A Biocompatible β-Cyclodextrin Inclusion Complex Containing Natural Extracts: A Promising Antibiofilm Agent

Obaydah Abd Alkader Alabrahim[1], Mostafa Fytory[1,2], Ahmed M. Abou-Shanab[3], Jude Lababidi[1], Wolfgang Fritzsche[4], Nagwa El-Badri[3*], Hassan Mohamed El-Said Azzazy[1,4*]

[1]Department of Chemistry, School of Sciences & Engineering, The American University in Cairo, AUC Avenue, P.O. Box 74, New Cairo 11835, Egypt.

[2]Material Science and Nanotechnology Department, Faculty of Postgraduate Studies for Advanced Sciences (PSAS), Beni-Suef University, 62511, Beni-Suef.

[3]Center of Excellence for Stem Cells and Regenerative Medicine, Zewail City of Science and Technology, Giza, 12578, Egypt.

[4]Department of Nanobiophotonics, Leibniz Institute of Photonic Technology, Jena, Germany.

***Corresponding authors:**

**Distinguished University Prof. Hassan M. E. Azzazy**

Department of Chemistry, School of Sciences & Engineering, The American University in Cairo, AUC Avenue, P.O. Box 74, New Cairo 11835, Egypt.

Tel: 00 202 2615 2559

Email: hazzazy@aucegypt.edu

**Prof. Nagwa El-Badri**

Center of Excellence for Stem Cells and Regenerative Medicine, Zewail City of Science and Technology, Giza, 12578, Egypt

Email: nelbadri@zewailcity.edu.eg

**Table of contents:**

**Figure S1.** SEM images of (**a**) βCD and (**b**) BOS-βCD inclusion complex. The βCD field demonstrated large ovoid crystals alongside rectangular particles, whereas the particles of BOS-βCD inclusion complex were presented with significant reduction in size and substantial morphological alterations developing several aggregates as well. These observations suggest the potential complexation formation of BOS-βCD inclusion complex, possibly involving an amorphous product interacting with other components in the established complex.

**Figure S2.** FTIR spectra for BOS, βCD, and BOS-βCD inclusion complex.

**Figure S3.** $^{1}$H NMR spectrum and stacked H1 Sub-spectra of BOS EO.

**Figure S4.** $^{1}$H NMR spectrum and stacked H1 Sub-spectra of βCD.

**Figure S5.** $^{1}$H NMR spectrum and stacked H1 Sub-spectra of BOS-βCD.

**Supplementary Table 1.** Chemical shifts ($\delta$) for βCD and BOS-βCD and differences in chemical shift ($\Delta\delta$)

**Figure S6.** Microdilution assays for MICs determination of BOS EO & BOS-βCD complex (Alamar Blue).

**Figure S7I.** Assay of Biofilm formation prevention against *S. aureus*.

**Figure S7II.** Assay of Biofilm formation prevention against *P. putida*.

**Figure S7III.** Assay of Biofilm formation prevention against *E. coli*.

**Figure S7IV.** Assay of Biofilm formation prevention against *B. subtilis*.

**Figure S8I.** Assay of established biofilm disruption against *S. aureus*.

**Figure S8II.** Assay of established biofilm disruption against *P. putida*.

**Figure S8III.** Assay of established biofilm disruption against *E. coli*.

**Figure S8IV.** Assay of established biofilm disruption against *B. subtilis*.

**Supplementary Figure S1.**

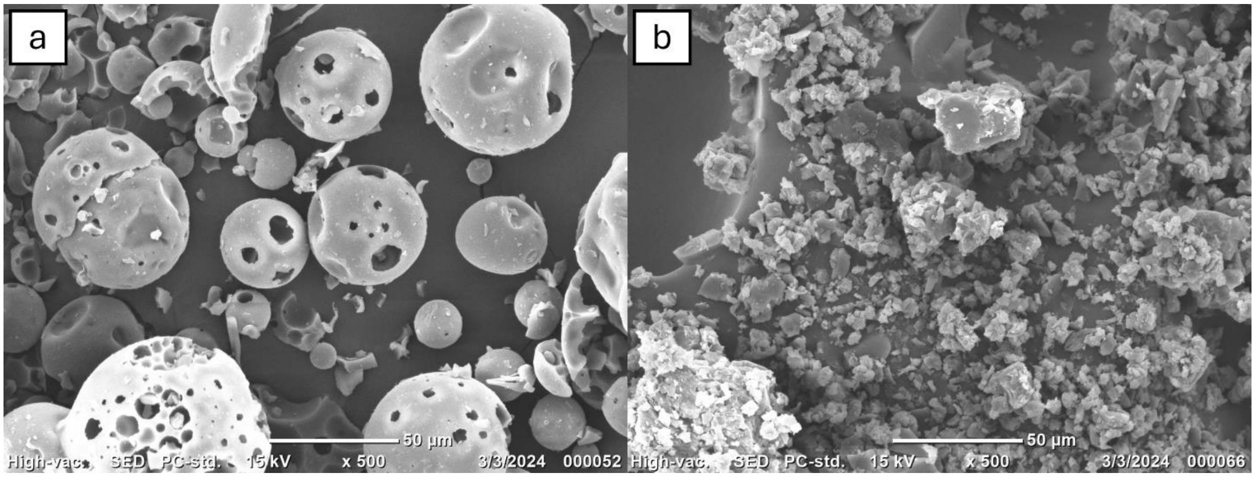


**Figure S1.** SEM images of (**a**) βCD and (**b**) BOS-βCD inclusion complex. The βCD field demonstrated large ovoid crystals alongside rectangular particles, whereas the particles of BOS-βCD inclusion complex were presented with significant reduction in size and substantial morphological alterations developing several aggregates as well. These observations suggest the potential complexation formation of BOS-βCD inclusion complex, possibly involving an amorphous product interacting with other components in the established complex.

**Supplementary Figure S2.**

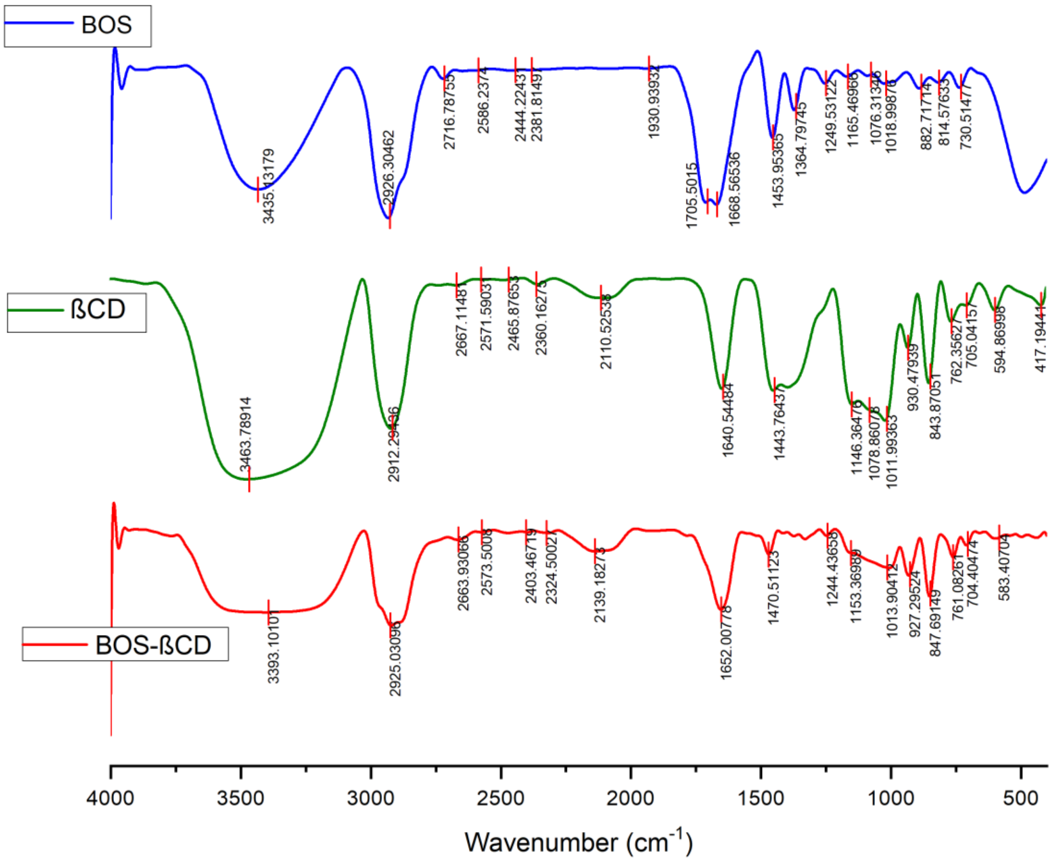


**Figure S2.** FTIR spectra for BOS, βCD, and BOS-βCD inclusion complex.

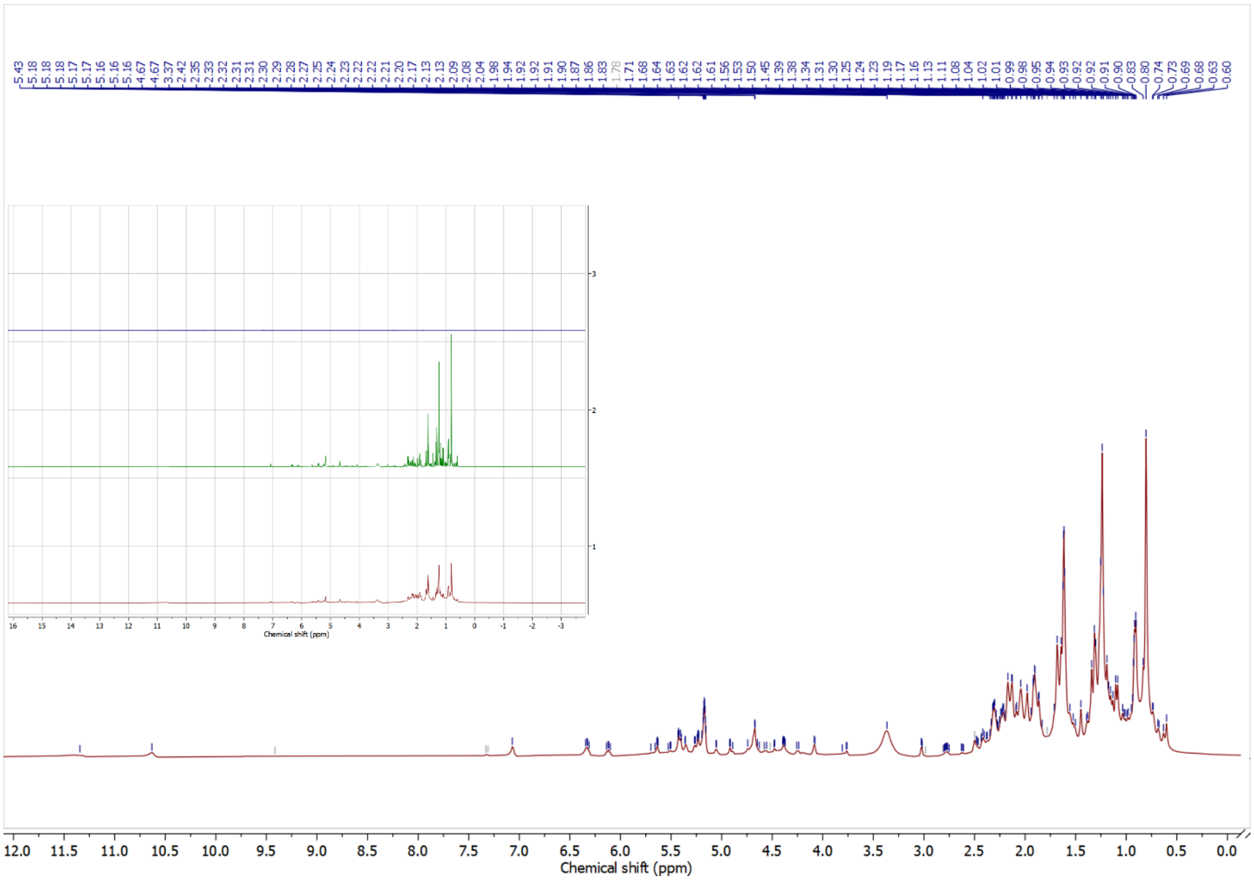


**Figure S3.** $^{1}$H NMR spectrum and stacked H1 Sub-spectra of BOS EO.

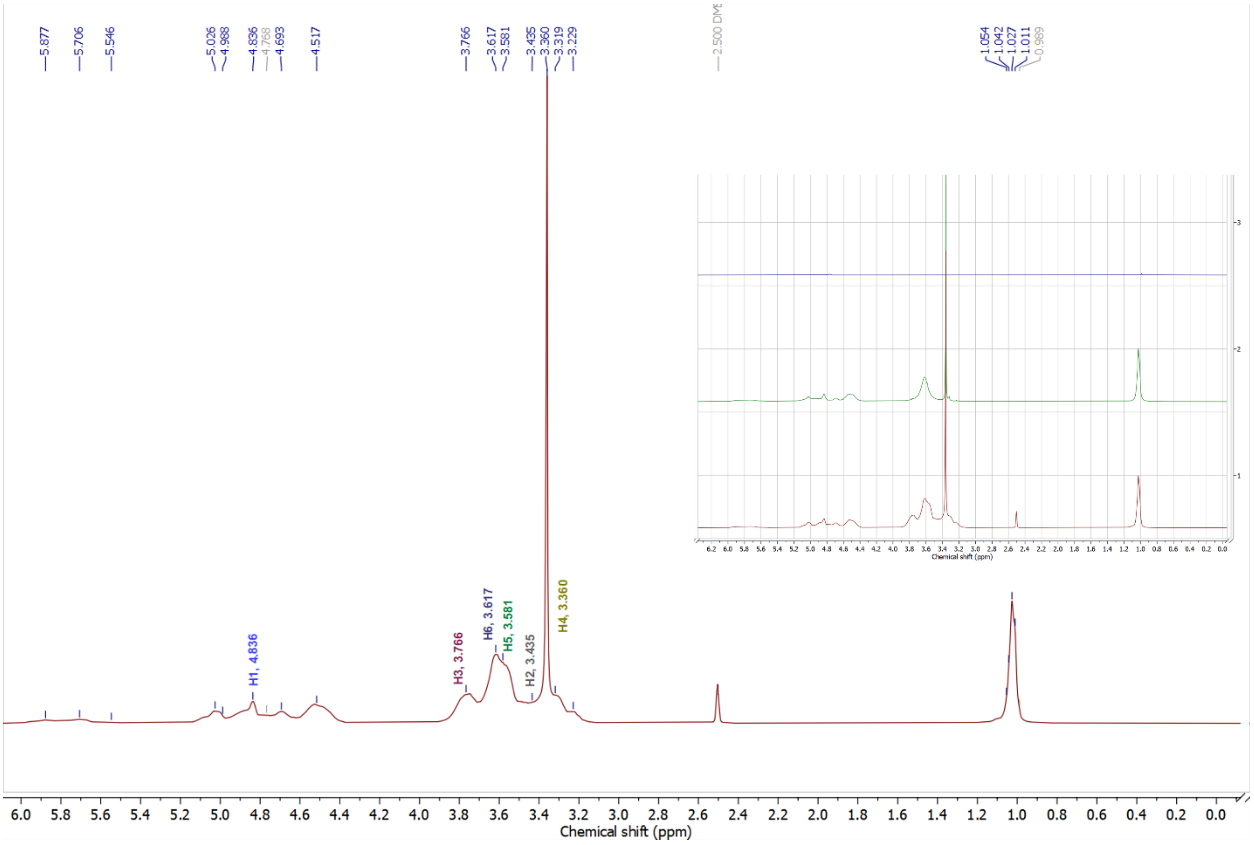


**Figure S4.** $^{1}$H NMR spectrum and stacked H1 Sub-spectra of βCD.

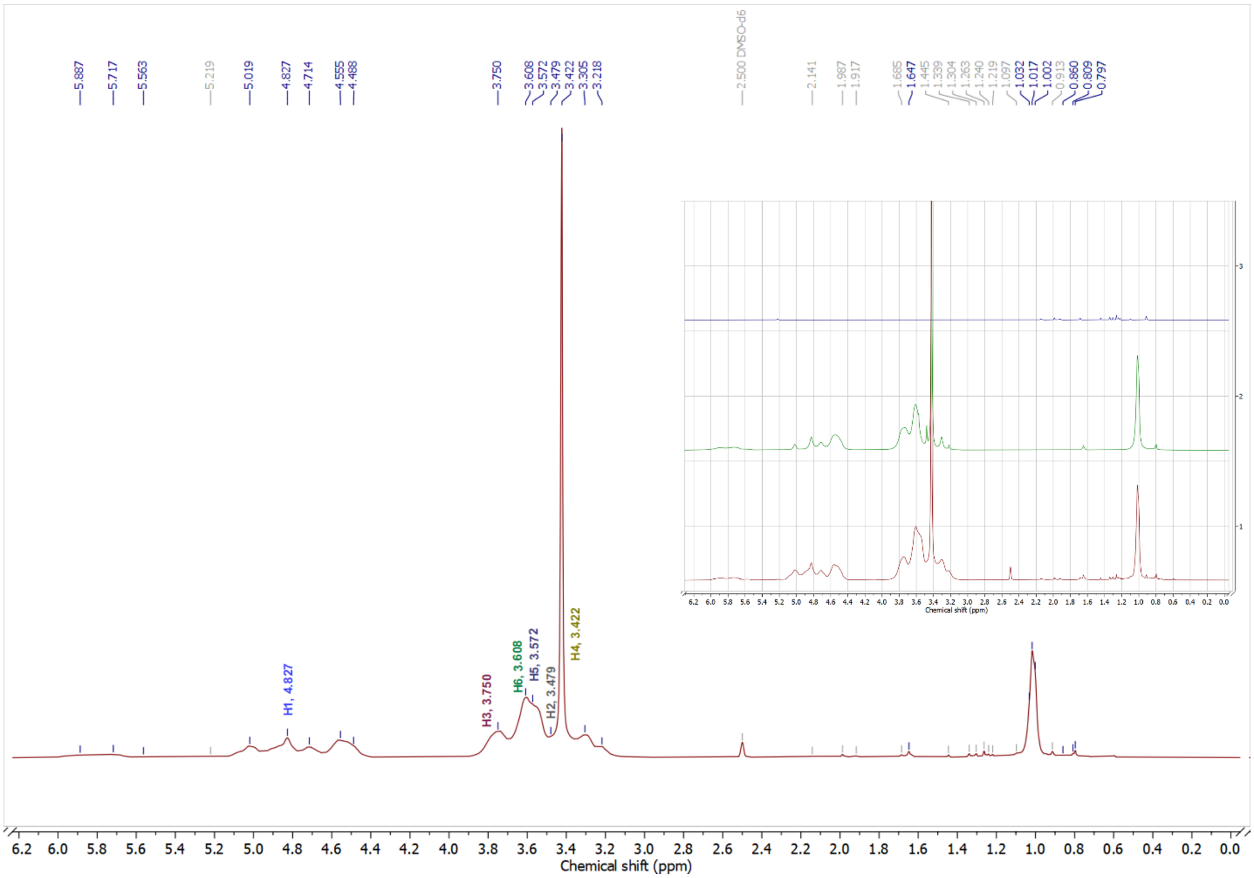


**Figure S5.** $^{1}$H NMR spectrum and stacked H1 Sub-spectra of BOS-βCD.

**Supplementary Table 1.** Chemical shifts (*δ*) for βCD and BOS-βCD and differences in chemical shift (Δ*δ*)

| Proton no. | βCD (*δ*/ppm) | BOS- βCD (*δ*/ppm) | Δ*δ* BOS-βCD/βCD (Δ*δ*/ppm) |
|---|---|---|---|
| H1 | 4.836 | 4.827 | 0.009 |
| H2 | 3.435 | 3.479 | -0.044 |
| H3 | 3.766 | 3.750 | 0.016 |
| H4 | 3.360 | 3.422 | -0.062 |
| H5 | 3.581 | 3.572 | 0.009 |
| H6 | 3.617 | 3.608 | 0.009 |

**Supplementary Figure S6.**

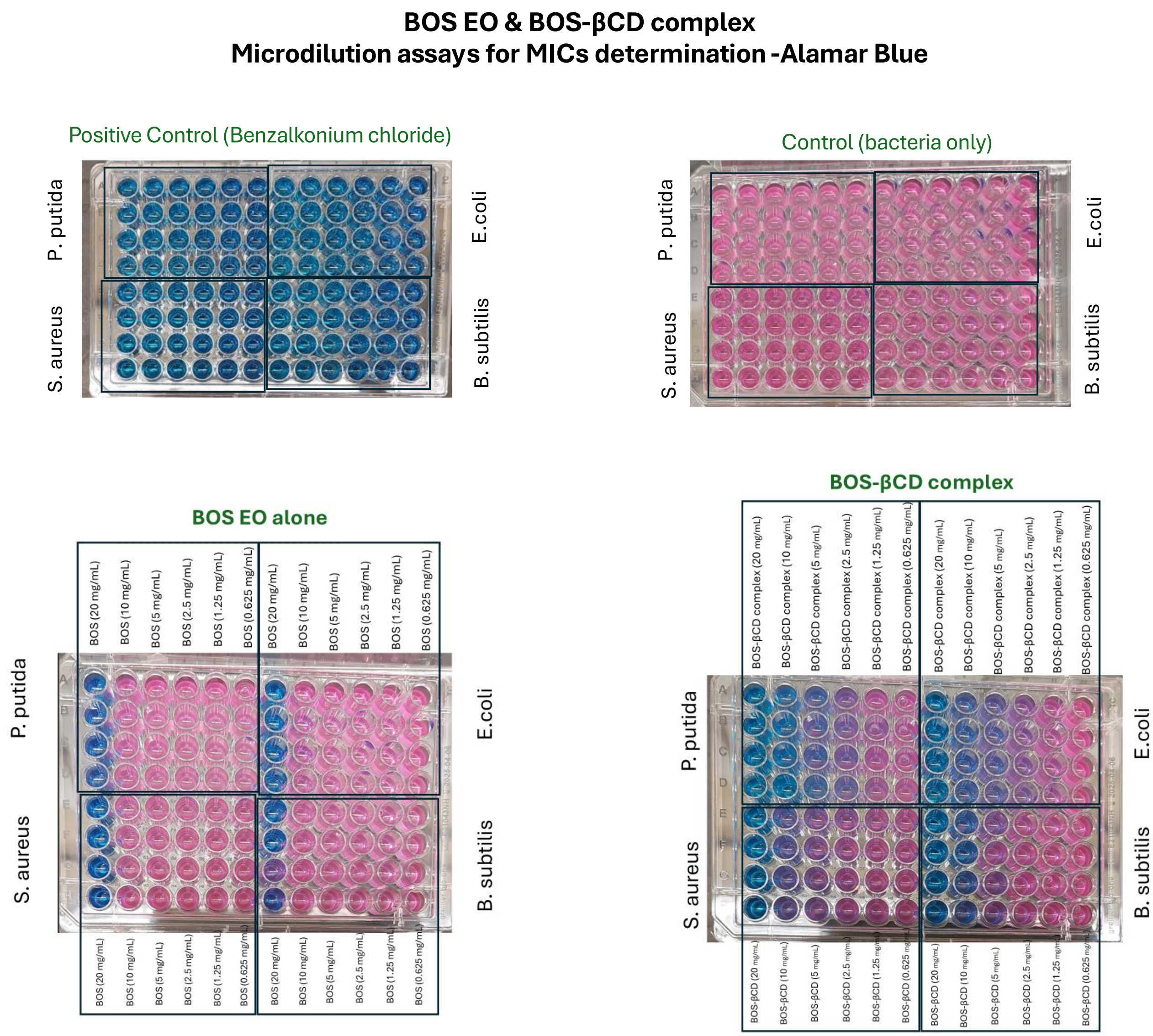


**Figure S6.** Microdilution assays for MICs determination of BOS EO & BOS-βCD complex (Alamar Blue).

**Supplementary Figure S7I.**

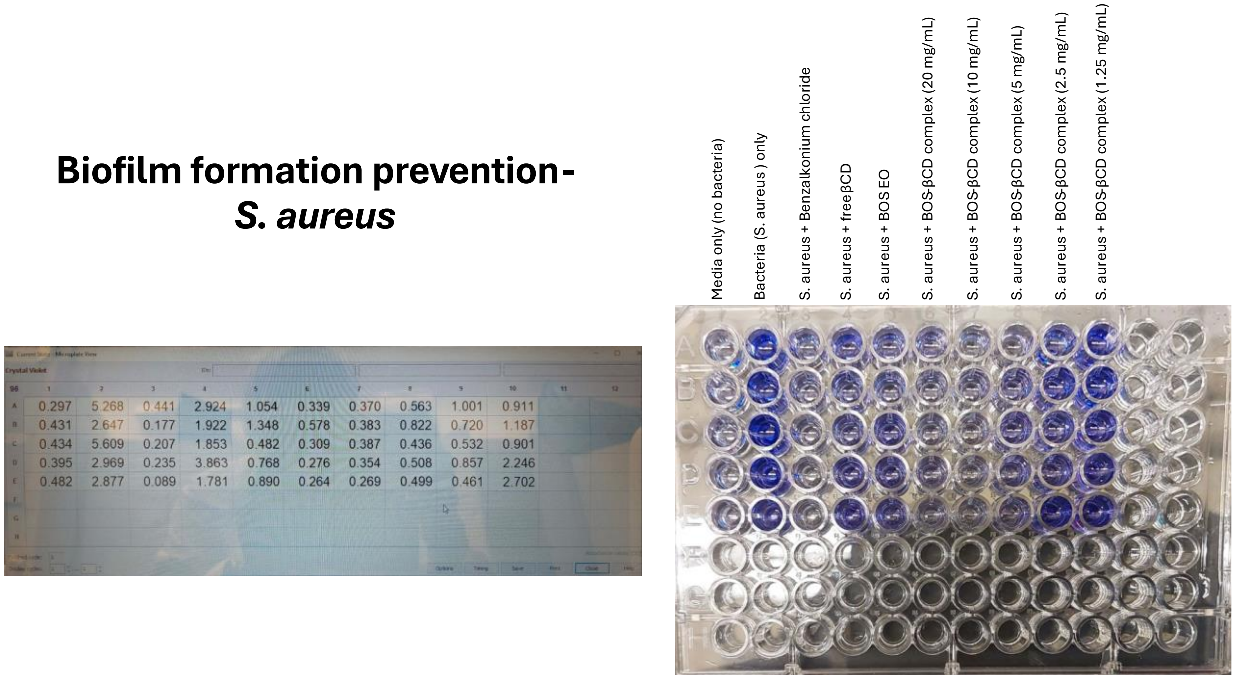


| 96 | 1 | 2 | 3 | 4 | 5 | 6 | 7 | 8 | 9 | 10 | 11 | 12 |
|---|---|---|---|---|---|---|---|---|---|---|---|---|
| A | 0.297 | 5.268 | 0.441 | 2.924 | 1.054 | 0.339 | 0.370 | 0.563 | 1.001 | 0.911 | | |
| B | 0.431 | 2.647 | 0.177 | 1.922 | 1.348 | 0.578 | 0.383 | 0.822 | 0.720 | 1.187 | | |
| C | 0.434 | 5.609 | 0.207 | 1.853 | 0.482 | 0.309 | 0.387 | 0.436 | 0.532 | 0.901 | | |
| D | 0.395 | 2.969 | 0.235 | 3.863 | 0.768 | 0.276 | 0.354 | 0.508 | 0.857 | 2.246 | | |
| E | 0.482 | 2.877 | 0.089 | 1.781 | 0.890 | 0.264 | 0.269 | 0.499 | 0.461 | 2.702 | | |
| F | | | | | | | | | | | | |
| G | | | | | | | | | | | | |
| H | | | | | | | | | | | | |

**Figure S7I.** Assay of Biofilm formation prevention against *S. aureus*.

**Supplementary Figure S7II.**

**Biofilm formation prevention - *P. Putida***

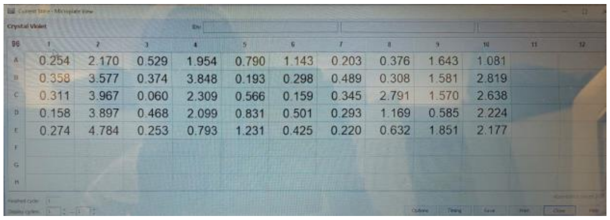

Crystal Violet

| 96 | 1 | 2 | 3 | 4 | 5 | 6 | 7 | 8 | 9 | 10 | 11 | 12 |
|---|---|---|---|---|---|---|---|---|---|---|---|---|
| A | 0.254 | 2.170 | 0.529 | 1.954 | 0.790 | 1.143 | 0.203 | 0.376 | 1.643 | 1.081 | | |
| B | 0.358 | 3.577 | 0.374 | 3.848 | 0.193 | 0.298 | 0.489 | 0.308 | 1.581 | 2.819 | | |
| C | 0.311 | 3.967 | 0.060 | 2.309 | 0.566 | 0.159 | 0.345 | 2.791 | 1.570 | 2.638 | | |
| D | 0.158 | 3.897 | 0.468 | 2.099 | 0.831 | 0.501 | 0.293 | 1.169 | 0.585 | 2.224 | | |
| E | 0.274 | 4.784 | 0.253 | 0.793 | 1.231 | 0.425 | 0.220 | 0.632 | 1.851 | 2.177 | | |
| F | | | | | | | | | | | | |
| G | | | | | | | | | | | | |
| H | | | | | | | | | | | | |

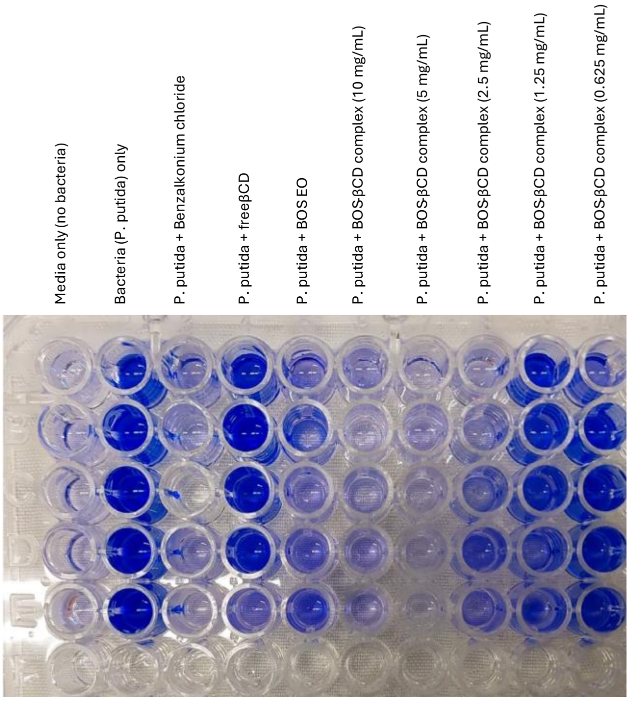


**Figure S7II.** Assay of Biofilm formation prevention against *P. putida*.

**Supplementary Figure S7III.**

**Biofilm formation prevention - *E. coli***

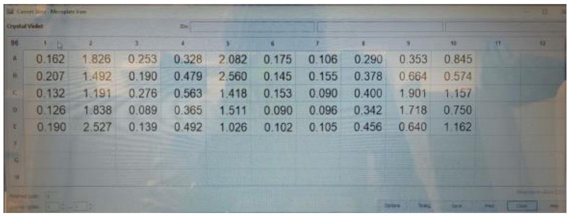

Crystal Violet

| 96 | 1 | 2 | 3 | 4 | 5 | 6 | 7 | 8 | 9 | 10 |
|---|---|---|---|---|---|---|---|---|---|---|
| A | 0.162 | 1.826 | 0.253 | 0.328 | 2.082 | 0.175 | 0.106 | 0.290 | 0.353 | 0.845 |
| B | 0.207 | 1.492 | 0.190 | 0.479 | 2.560 | 0.145 | 0.155 | 0.378 | 0.664 | 0.574 |
| C | 0.132 | 1.191 | 0.276 | 0.563 | 1.418 | 0.153 | 0.090 | 0.400 | 1.901 | 1.157 |
| D | 0.126 | 1.838 | 0.089 | 0.365 | 1.511 | 0.090 | 0.096 | 0.342 | 1.718 | 0.750 |
| E | 0.190 | 2.527 | 0.139 | 0.492 | 1.026 | 0.102 | 0.105 | 0.456 | 0.640 | 1.162 |

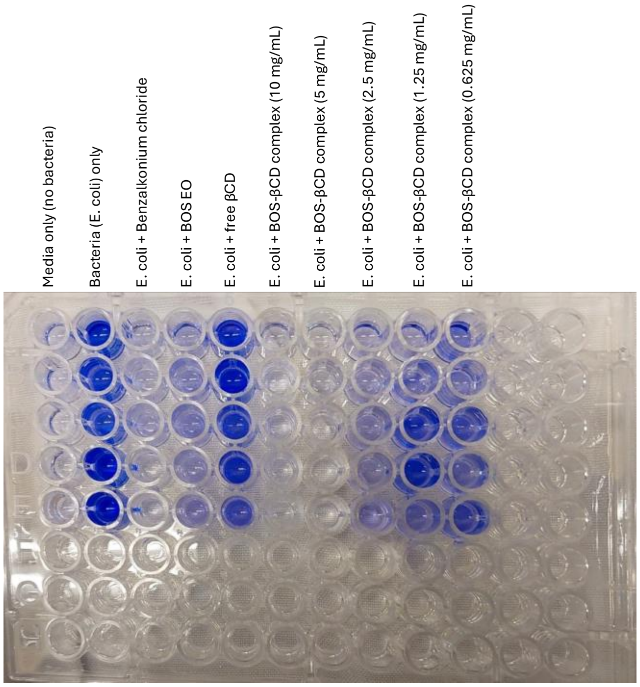


**Figure S7III.** Assay of Biofilm formation prevention against *E. coli*.

**Supplementary Figure S7IV.**

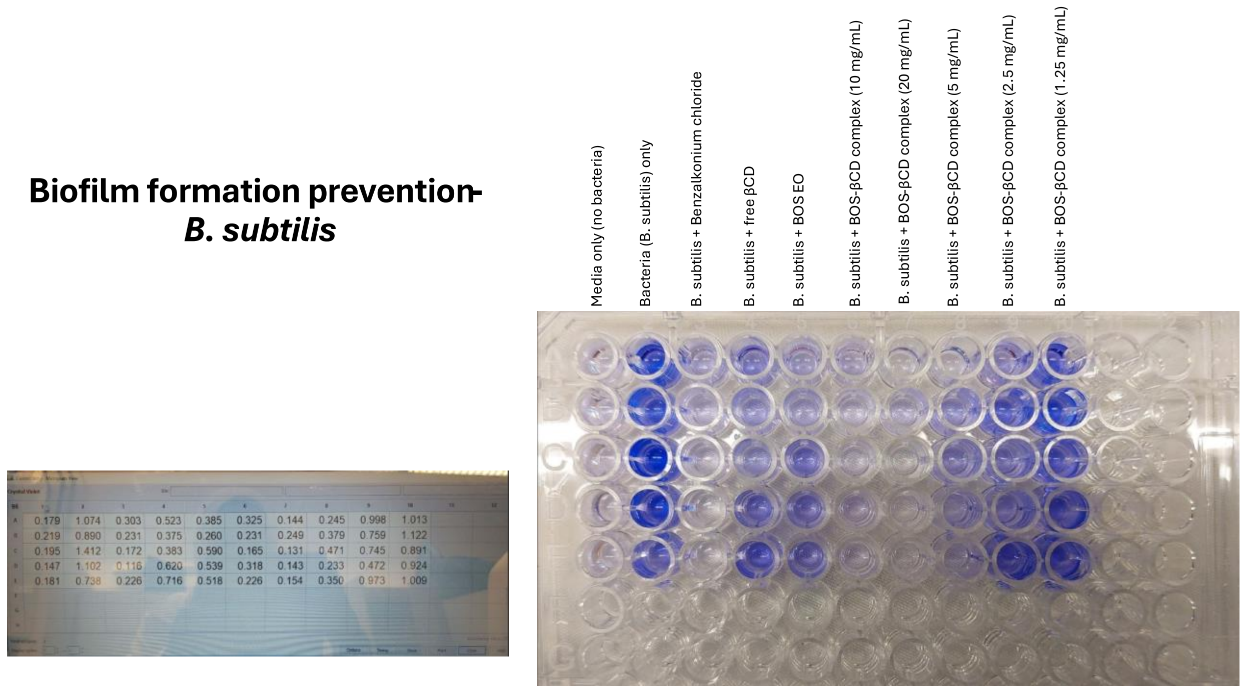


| | 1 | 2 | 3 | 4 | 5 | 6 | 7 | 8 | 9 | 10 | 11 | 12 |
|---|---|---|---|---|---|---|---|---|---|---|---|---|
| A | 0.179 | 1.074 | 0.303 | 0.523 | 0.385 | 0.325 | 0.144 | 0.245 | 0.998 | 1.013 | | |
| B | 0.219 | 0.890 | 0.231 | 0.375 | 0.260 | 0.231 | 0.249 | 0.379 | 0.759 | 1.122 | | |
| C | 0.195 | 1.412 | 0.172 | 0.383 | 0.590 | 0.165 | 0.131 | 0.471 | 0.745 | 0.891 | | |
| D | 0.147 | 1.102 | 0.116 | 0.620 | 0.539 | 0.318 | 0.143 | 0.233 | 0.472 | 0.924 | | |
| E | 0.181 | 0.738 | 0.226 | 0.716 | 0.518 | 0.226 | 0.154 | 0.350 | 0.973 | 1.009 | | |
| F | | | | | | | | | | | | |
| G | | | | | | | | | | | | |
| H | | | | | | | | | | | | |

**Figure S7IV.** Assay of Biofilm formation prevention against *B. subtilis*.

**Supplementary Figure S8I.**

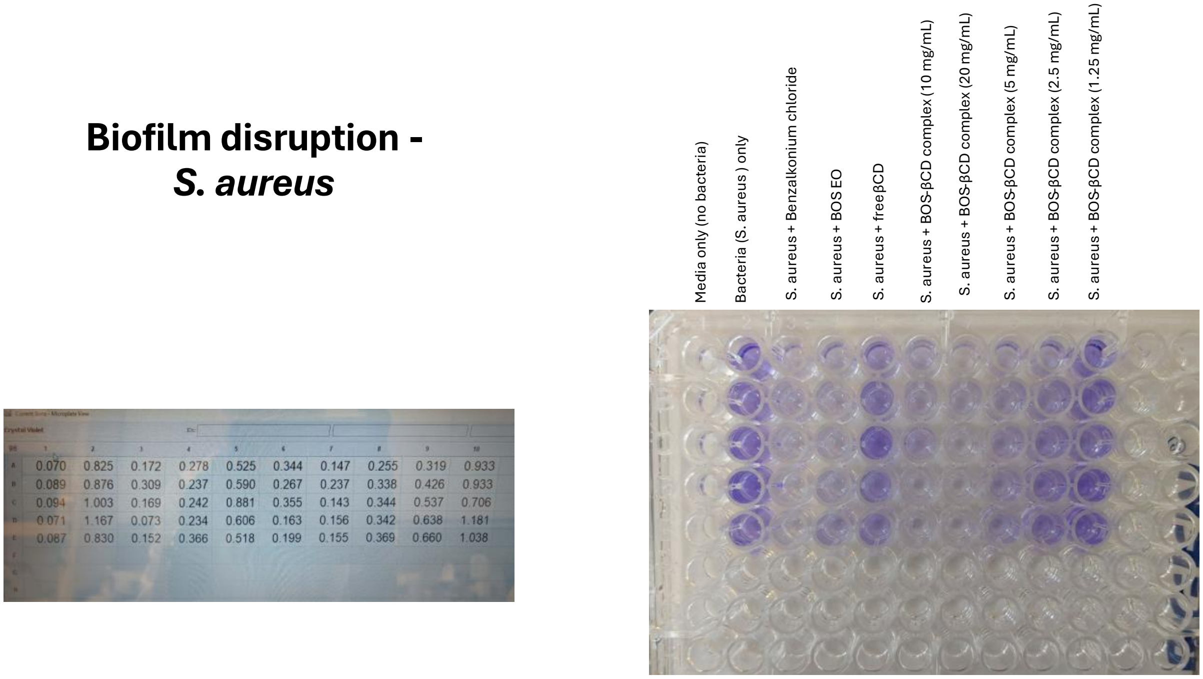


| 96 | 1 | 2 | 3 | 4 | 5 | 6 | 7 | 8 | 9 | 10 |
|---|---|---|---|---|---|---|---|---|---|---|
| A | 0.070 | 0.825 | 0.172 | 0.278 | 0.525 | 0.344 | 0.147 | 0.255 | 0.319 | 0.933 |
| B | 0.089 | 0.876 | 0.309 | 0.237 | 0.590 | 0.267 | 0.237 | 0.338 | 0.426 | 0.933 |
| C | 0.094 | 1.003 | 0.169 | 0.242 | 0.881 | 0.355 | 0.143 | 0.344 | 0.537 | 0.706 |
| D | 0.071 | 1.167 | 0.073 | 0.234 | 0.606 | 0.163 | 0.156 | 0.342 | 0.638 | 1.181 |
| E | 0.087 | 0.830 | 0.152 | 0.366 | 0.518 | 0.199 | 0.155 | 0.369 | 0.660 | 1.038 |
| F | | | | | | | | | | |
| G | | | | | | | | | | |
| H | | | | | | | | | | |

**Figure S8I.** Assay of established biofilm disruption against *S. aureus*.

**Supplementary Figure S8II.**

## Biofilm disruption - *P.Putida*

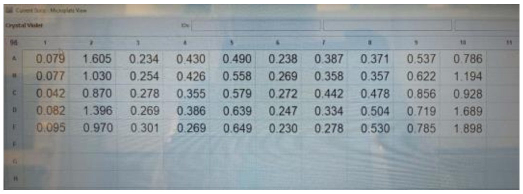

Crystal Violet

| 96 | 1 | 2 | 3 | 4 | 5 | 6 | 7 | 8 | 9 | 10 | 11 |
|---|---|---|---|---|---|---|---|---|---|---|---|
| A | 0.079 | 1.605 | 0.234 | 0.430 | 0.490 | 0.238 | 0.387 | 0.371 | 0.537 | 0.786 | |
| B | 0.077 | 1.030 | 0.254 | 0.426 | 0.558 | 0.269 | 0.358 | 0.357 | 0.622 | 1.194 | |
| C | 0.042 | 0.870 | 0.278 | 0.355 | 0.579 | 0.272 | 0.442 | 0.478 | 0.856 | 0.928 | |
| D | 0.082 | 1.396 | 0.269 | 0.386 | 0.639 | 0.247 | 0.334 | 0.504 | 0.719 | 1.689 | |
| E | 0.095 | 0.970 | 0.301 | 0.269 | 0.649 | 0.230 | 0.278 | 0.530 | 0.785 | 1.898 | |
| F | | | | | | | | | | | |
| G | | | | | | | | | | | |
| H | | | | | | | | | | | |

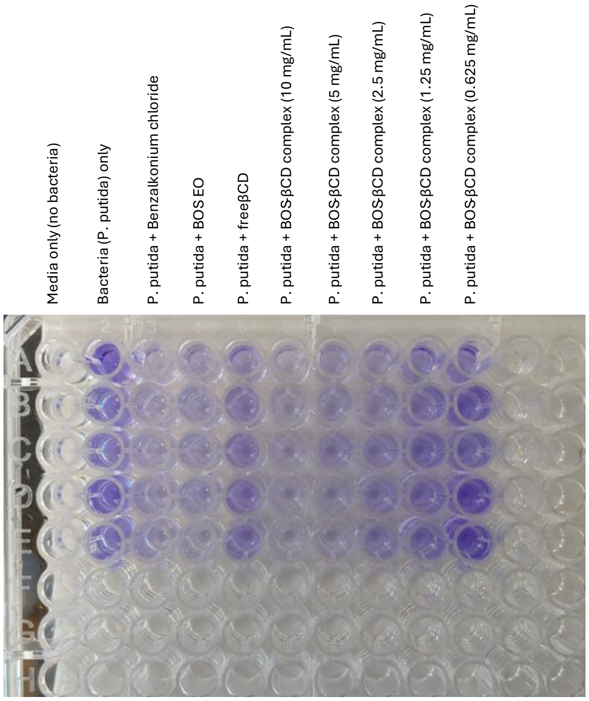


**Figure S8II.** Assay of established biofilm disruption against *P. putida*.

**Supplementary Figure S8III.**

## Biofilm disruption - *E. coli*

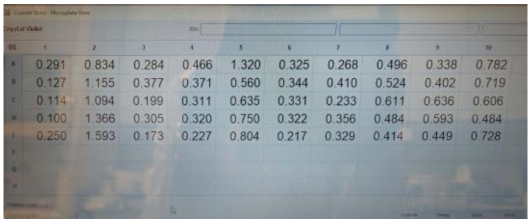

| 96 | 1 | 2 | 3 | 4 | 5 | 6 | 7 | 8 | 9 | 10 |
|---|---|---|---|---|---|---|---|---|---|---|
| A | 0.291 | 0.834 | 0.284 | 0.466 | 1.320 | 0.325 | 0.268 | 0.496 | 0.338 | 0.782 |
| B | 0.127 | 1.155 | 0.377 | 0.371 | 0.560 | 0.344 | 0.410 | 0.524 | 0.402 | 0.719 |
| C | 0.114 | 1.094 | 0.199 | 0.311 | 0.635 | 0.331 | 0.233 | 0.611 | 0.636 | 0.606 |
| D | 0.100 | 1.366 | 0.305 | 0.320 | 0.750 | 0.322 | 0.356 | 0.484 | 0.593 | 0.484 |
| E | 0.250 | 1.593 | 0.173 | 0.227 | 0.804 | 0.217 | 0.329 | 0.414 | 0.449 | 0.728 |

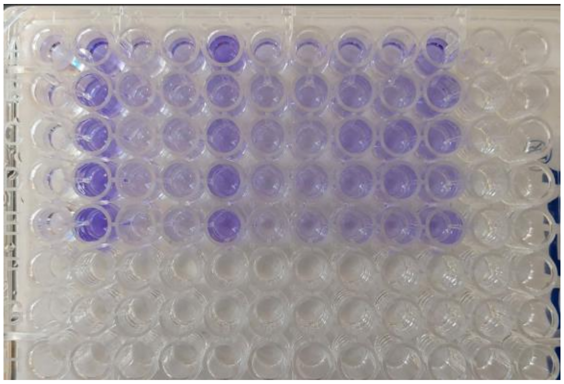


**Figure S8III.** Assay of established biofilm disruption against *E. coli*.

**Supplementary Figure S8IV.**

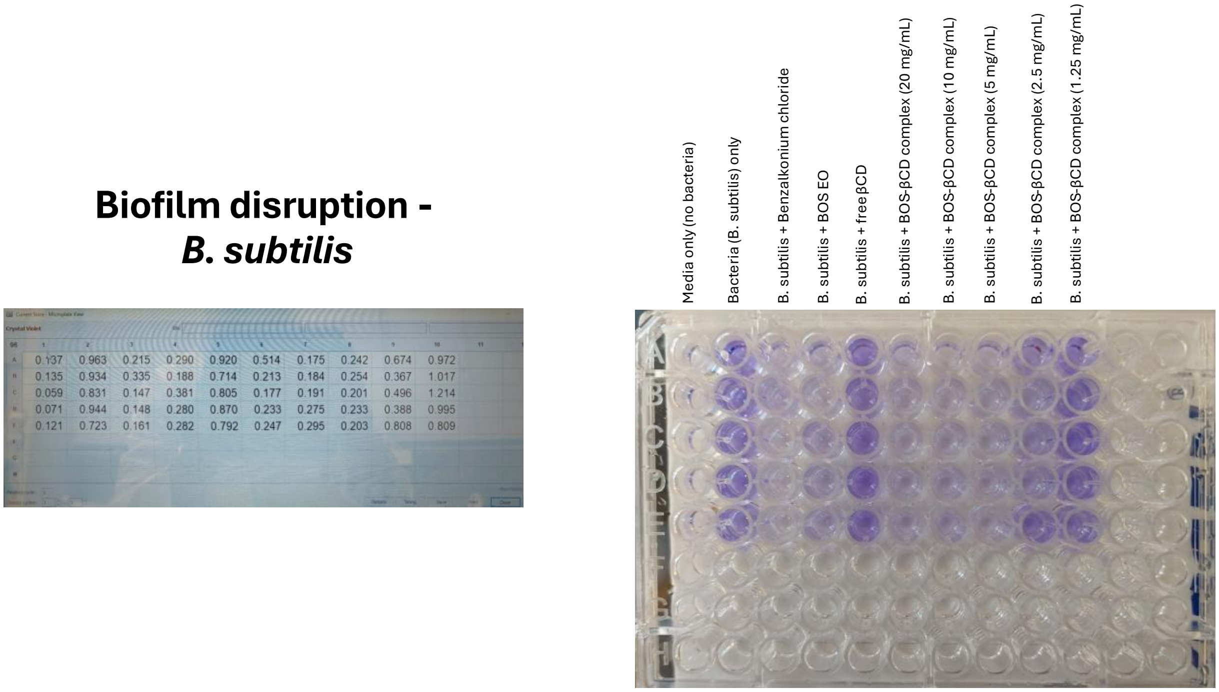


| 96 | 1 | 2 | 3 | 4 | 5 | 6 | 7 | 8 | 9 | 10 | 11 |
|---|---|---|---|---|---|---|---|---|---|---|---|
| A | 0.137 | 0.963 | 0.215 | 0.290 | 0.920 | 0.514 | 0.175 | 0.242 | 0.674 | 0.972 | |
| B | 0.135 | 0.934 | 0.335 | 0.188 | 0.714 | 0.213 | 0.184 | 0.254 | 0.367 | 1.017 | |
| C | 0.059 | 0.831 | 0.147 | 0.381 | 0.805 | 0.177 | 0.191 | 0.201 | 0.496 | 1.214 | |
| D | 0.071 | 0.944 | 0.148 | 0.280 | 0.870 | 0.233 | 0.275 | 0.233 | 0.388 | 0.995 | |
| E | 0.121 | 0.723 | 0.161 | 0.282 | 0.792 | 0.247 | 0.295 | 0.203 | 0.808 | 0.809 | |
| F | | | | | | | | | | | |
| G | | | | | | | | | | | |
| H | | | | | | | | | | | |

**Figure S8IV.** Assay of established biofilm disruption against *B. subtilis*.
